# Evidence for validity of the Swedish self-rated 36-item version of the World Health Organization Disability Assessment Schedule 2.0 (WHODAS 2.0) in patients with mental disorders: a multi-centre cross-sectional study using Rasch analysis

**DOI:** 10.1186/s41687-022-00449-8

**Published:** 2022-05-08

**Authors:** Cecilia Svanborg, Ahmed Amer, Axel Nordenskjöld, Mia Ramklint, Per Söderberg, Stefan Tungström, Ylva Ginsberg, Liselotte Hermansson

**Affiliations:** 1 Department of Clinical Neuroscience, Centre for Psychiatry Research, Karolinska Institutet, Stockholm, Sweden; 2 University Health Care Research Center, Faculty of Medicine and Health, Örebro University, Örebro, Sweden; 3 Department of Neuroscience, Psychiatry, Uppsala University, Uppsala, Sweden; 4 Psychiatric Research and Development Department, Säter, Sweden

**Keywords:** Psychiatry, Mental health, Disability, Assessment, Validity, Reliability, Psychometric

## Abstract

**Background:**

The World Health Organization Disability Assessment Schedule 2.0 (WHODAS 2.0) is a generic instrument for the assessment of functioning in six domains, resulting in a total health-related disability score. The aim of this study was to investigate the psychometric properties of the Swedish-language version of the self-rated 36-item version in psychiatric outpatients with various common psychiatric diagnoses using Rasch analysis. A secondary aim was to explore the correlation between two methods of calculating overall scores to guide clinical practice: the WHODAS simple (summative) model and the WHODAS complex (weighted) model.

**Methods:**

Cross-sectional data from 780 Swedish patients with various mental disorders were evaluated by Rasch analysis according to the partial credit model. Bivariate Pearson correlations between the two methods of calculating overall scores were explored.

**Results:**

Of the 36 items, 97% (35 items) were within the recommended range of infit mean square; only item D4.5 (Sexual activities) indicated misfit (infit mean square 1.54 logits). Rating scale analysis showed a short distance between severity levels and disordered thresholds. The two methods of calculating overall scores were highly correlated (0.89–0.99).

**Conclusions:**

The self-administered WHODAS 2.0 fulfilled several aspects of validity according to Rasch analysis and has the potential to be a useful tool for the assessment of functioning in psychiatric outpatients. The internal structure of the instrument was satisfactorily valid and reliable at the level of the total score but demonstrated problems at the domain level. We suggest rephrasing the item *Sexual activities* and revising the rating scale categories. The WHODAS simple model is easier to use in clinical practice and our results indicate that it can differentiate function among patients with moderate psychiatric disability, whereas Rasch scaled scores are psychometrically more precise even at low disability levels. Further investigations of different scoring models are warranted.

**Supplementary Information:**

The online version contains supplementary material available at 10.1186/s41687-022-00449-8.

## Introduction

Mental disorders constitute a large proportion of the disability in society, which is commonly explained by their early onset and high incidence rates [1]. Furthermore, mental disorders often have a chronic course, with waxing and waning levels of symptoms and impairment in many areas of life. Managing people’s functional disability caused by mental disorders is therefore one of the greatest challenges in health care. Functioning is defined as an individual’s ability to manage relations, work tasks, home chores and other tasks. A person’s functional level depends on the severity of his or her symptoms, personal resources and ability to handle the illness, as well as contextual factors in society. In health care, assessments of functioning may be useful for many reasons: to determine patients’ need for support, measure treatment effects, monitor changes over time and predict treatment outcomes. In order to meaningfully conduct such assessments, valid and reliable measures of functioning are needed [2].

The World Health Organization Disability Assessment Schedule (WHODAS) 2.0 was developed by an international working group to create a generic tool for measuring patients’ perspectives on disability and functioning [3] based on the International Classification of Functioning, Disability and Health (ICF) [4]. In ICF, disability and functioning are conceptualized as interactions between the individual’s health status, activity and participation, and the context, i.e. environmental and personal factors. Positive and neutral aspects of those interactions are referred to as functioning, while negative aspects are referred to as disability. The WHODAS 2.0 measures an individual’s subjective functioning and disability in daily life during the past 30 days in relation to his or her current health condition in six domains: Cognition (understanding and communicating); Mobility (moving and getting around); Self-care (hygiene, dressing, eating and staying alone); Getting along (interacting with other people); Life activities (a = domestic responsibilities, and b = work and school); and Participation (joining in community and leisure time activities). It has been translated, validated and used in many health care fields [5]. A Swedish version of WHODAS 2.0 was created in accordance with WHO guidelines by a working group under the Swedish National Board of Health and Welfare [6]. In the field of mental disorders, the latest version of the Diagnostic and Statistical Manual of Mental Disorders (DSM-5) [7] has replaced the formerly recommended Global Assessment of Functioning scale [8], with the WHODAS 2.0 as the suggested method for disability assessment.

Before we implement the WHODAS 2.0 into Swedish psychiatry practice, we need to gather evidence for the validity of the Swedish version of the instrument in its intended context of use [9]. One of the characteristics of a test is its rating scale and method of calculating an overall score. The WHODAS 2.0 manual presents two methods of calculation: the simple model and the complex model. The simple model is merely a summation of the raw scores given and can easily be converted to an overall percentage of possible scores using the scoring template available at the WHO website [10]. The complex model is based on item response theory (IRT) and considers multiple levels of difficulty for each item [11]; however, no information is available from the manual or the original paper about which IRT model is the basis of the scoring. According to the complex scoring model in the WHODAS 2.0 manual [3], the rating scale categories should be collapsed from five to three (categories 1 and 2 become category 1, categories 3 and 4 become category 2, and category 5 becomes category 3) for 19 out of the 36 items. No information is available on how the decision to collapse these rating categories was made and why these items were chosen. The WHODAS 2.0 manual provides an algorithm for computing an overall score according to this model. However, whether a difference in the overall score exists depending on the scoring model used has not been established. The complex model may require more time than the simple model for the clinician using the instrument. Thus, the scoring models require further examination in order that clinicians can receive more guidance on which one to use.

Several international validation studies have been performed on the WHODAS 2.0 using classical test theory (CTT) [5]. Modern test theory, i.e. IRT, such as Rasch analysis, allows analyses that also provide information about rating scales, items and item bias between subgroups [12]. Item and individual characteristics of the 36-item WHODAS 2.0 have been evaluated using Rasch analysis in international populations with spinal cord injury [13], multiple sclerosis [14], stroke [15] and osteoarthritis [16]. Some studies have examined the self-administrated version [14, 17], but the majority explored the interviewer-administrated version, and only two of the studies involved patients with mental disorders, namely, schizophrenia and drug addiction with comorbid mental disorders [18, 19]. These studies found support for the validity of the total scores in WHODAS 2.0 and its predecessor WHODAS II, but they also noted some misfit concerning domains and specific items. The use of both CTT and IRT has been suggested to be more informative than the use of only one of these methods [20]. Midhage et al. performed CTT analyses on WHODAS 2.0 data from Swedish patients with mental disorders [21]. The results showed good reliability (Cronbach’s alpha values for domains were between 0.70–0.90, and test–retest reliability of the total score was ICC 0.83) and convergent validity (Pearson correlation coefficient of 0.77 between the WHODAS 2.0 and the Sheehan Disability Scale). However, this study provided no information about other psychometric properties such as item fit, bias or rating scale functioning [21, 22]. Therefore, further investigation of these properties by Rasch analysis on the Swedish version of the WHODAS 2.0 in patients with mental disorders is important.

The aim of this study was to investigate the psychometric properties of the Swedish self-rated 36-item version of the WHODAS 2.0 in a psychiatric outpatient population with various common psychiatric diagnoses by testing the instrument’s internal structure by means of Rasch analysis. A secondary aim was to explore the correlation between two methods of calculating overall scores to guide clinical practice.

## Methods

A multi-centre cross-sectional design was used. The Regional Ethics Review board in Uppsala and in Stockholm approved all procedures (approval number 2014/1489-31/4 and 2015/339, respectively).

### Participants and procedure

To obtain 99% confidence that the item calibration (item difficulty measure) is within ± ½ logit of its stable value, a minimum sample size of 243 is recommended [23]. To ensure the stability of item difficulty between participant groups (in other words, to limit item bias), it is recommended to have at least 100 participants per group [24]. Since we planned such analyses in groups based on sex (two groups), age (four groups) and diagnosis (seven groups), we required at least 700 participants. A cross-sectional convenience sample was chosen because no control over the recruitment process was possible. Patients at 20 psychiatric outpatient units in four regions in Central Sweden (Dalarna, Uppsala, Örebro, and Stockholm) were included. Data collection was conducted between December 2014 and December 2017. The inclusion criteria were the ability to read and understand Swedish. During a regular visit, the attending clinician provided written and oral information about the study and collected demographic and clinical information. In total, 837 patients agreed to participate in the study. All participants signed an informed consent form and completed the 36-item WHODAS 2.0 questionnaire.

In line with the recommendations in the WHODAS 2.0 manual, data with a maximum of two missing responses per subject, but no more than one missing response in any domain, were accepted for inclusion in the analyses. This led to 57 participants being omitted, and 780 remained in the final analyses. Each participant’s main diagnosis was reported by the clinician, or if the main diagnosis was missing or ambiguously reported, it was inferred from the type of clinic from which the participants were recruited. In 22 cases this was not possible, and these cases were thus without diagnosis. The mean age (standard deviation, SD) was 39.5 (15.7) years, and 65.6% of the participants were women. The distribution of participants with respect to sex, age group and diagnosis is reported in Table 1.

**Table 1:** Distribution of participants based on sex, age group and diagnosis, n = 780*

Age group	20–34 (n = 367)		35–49 (n = 185)		50–64 (n = 150)		65 + (n = 73)		Total (n = 775)	
Diagnosis:	F	M	F	M	F	M	F	M	F	M
Anxiety disorders (n = 61)	37	8	6	5	3	1	0	1	46	15
Affective disorders (n = 420)	124	38	79	34	48	34	41	22	292	128
Eating disorders (n = 45)	34	3	7	0	1	0	0	0	42	3
Psychotic disorders (n = 37)	3	5	5	4	7	6	3	4	18	19
Substance use disorders (n = 35)	2	5	1	5	3	18	0	1	6	29
ADHD and autism spectrum disorders (n = 142)	50	33	16	17	15	10	1	0	82	60
Other diagnosis (n = 13)	11	0	2	0	0	0	0	0	13	0
*Missing diagnosis (n* = *22)*	11	3	2	2	0	4	0	0	13	9
Total (n = 775)	272	95	118	67	77	73	45	28	512	263

^*^Demographic information not reported in five participants. (F = female, M = male.)

### Instrumentation

The WHODAS 2.0 is a generic standardized questionnaire available in 12-item, 12 + 24-item, and 36-item versions. For the 12 + 24 item version, the 12-item version is used to screen for problematic areas of functioning and, based on the responses to the 12 items, respondents may be given up to 24 additional questions from the 36-item version [11]. The WHODAS 2.0 measures difficulty in activity performance and participation through six domains: D1, Understanding and communicating; D2, Getting around; D3, Self-care; D4, Getting along with people; D5, Life activities; and D6, Participation in society. D5 (Life activities) is divided into two areas: D5a = Domestic responsibilities, and D5b = Work and school. In the 36-item version, the items that comprise the domains are distributed as follows: Cognition (D1.1–D1.6; six items), Mobility (D2.1–D2.5; five items), Self-care (D3.1–D3.4; four items), Getting along (D4.1–D4.5; five items), Life activities (D5.1–D5.4 [D5a]; D5.5–D5.8 [D5b]; both four items), and Participation (D.6.1–D6.8; eight items). The items are scored on a common five-point Likert scale ranging from 0 = *no difficulty* to 4 = *extreme difficulty or cannot do*. Thus, a higher score indicates a higher level of disability. The full version of the original WHODAS 2.0 can be found elsewhere [3].

The WHODAS 2.0 can be completed through self-report, interviewer administration, or proxy. For this study, the Swedish 36-item self-report version was used [6].

### Statistical analysis

Since each of the WHODAS domains can be used separately from the others or combined into a total summary score, we decided to run the analyses both for each domain separately and for all the domains together. Furthermore, in the WHODAS 2.0 complex scoring method there are two different rating scale structures (the collapsed three categories and the original five categories). This could be an indication that the rating scale structure has some problems. Hence, even though all items in WHODAS 2.0 share the same rating categories, as in other studies, we used the Rasch partial credit model to analyse each item separately [25, 26]. By using Rasch analysis, the data are evaluated against Rasch assumptions, such as unidimensionality (the assumption that all items reflect one single dimension, the latent variable, which is disability in our study). The recommended values reflect the hypothesis we test our data against. By investigating the psychometric properties of the instrument, we accumulate evidence for the validity of the WHODAS 2.0. More information about Rasch analysis can be found elsewhere [27].

With the original rating category order of WHODAS 2.0, a higher score indicates a higher level of disability. This is because more difficult items have a high measure (difficulty level in logits) whereas abler persons achieve a low measure. Since the output from the Rasch analysis is reported on the same scale for both items and persons, we changed the category order so that persons with greater ability received a higher measure. Therefore, before the analyses were performed, the order of the rating scale categories was reversed as follows: 0 = *extreme/cannot*, 1 = *severe*, 2 = *moderate*, 3 = *mild*, 4 = *no difficulty*.

Evidence for the validity of the WHODAS 2.0 was investigated based on six aspects:

**(I) Item fit:** The data were considered to usefully fit the Rasch model if at least 95% of the items (i.e. 34 of 36 items) had an infit mean square within the range 0.6–1.5 [28, 29]. *Infit* is more sensitive to the response pattern for items that are targeted on the person and vice versa [30]; therefore, it reflects whether the item hierarchy is similar for all responders. *Outfit* is more sensitive to the outlying responses, in other words, the performance of persons at a distance from the item’s location [27].

**(II) Unidimensionality:** The Rasch assumption is that items reflect only one main dimension. The principal component analysis (PCA) of residuals was used to investigate data against this assumption, that is, whether the unexplained part of the data (residuals) is random noise or demonstrates another meaningful dimension [31, 32]. Unidimensionality is supported when the variance explained by the main dimension is equal to or above 60% of the total variance [33] and the eigenvalue of the unexplained variance of the first contrast is less than 2 logits [31, 32]. Another indicator of unidimensionality is point-biserial correlation; a positive point-biserial correlation indicates that items contribute positively to the total raw score [34, 35]. A disattenuated correlation (correlation corrected for measurement error) indicates whether the subsets of items are correlated with each other under the same domain or measurement tool, which confirms unidimensionality [36]. A disattenuated correlation of approximately 1 indicates that the item subsets measure the same dimension (the same latent variable) [37]; the cut-off point for the disattenuated correlation was > 0.7 [38]. Another assumption was item local independency, meaning that items are independent from each other. That is, if one item is deleted from the instrument, this will not affect the other items [39]. Item independency was evaluated by measuring the correlation of residuals for two item pairs. Item local independency was assumed if the correlation coefficient was < 0.70 [40].

**(III) Reliability and separation of persons and items:** These were calculated based on person and item measures (in logits), respectively [33, 34]. Cronbach’s alpha was calculated based on raw scores to investigate the internal consistency; an alpha value > 0.80 was considered acceptable [41]. However, for instruments used in clinical evaluation, the recommended value is > 0.90 [41]. Item and person separation are additional reliability indices. *Item separation* indicates a difficulty hierarchy indicating how many strata of items can be differentiated by the respondents; low item separation indicates that the sample size is not large enough to confirm the item difficulty hierarchy. Low *person separation* with an appropriate sample size may indicate that the instrument is not sensitive enough to distinguish between persons based on their ability [42]. A separation value above 3 is recommended as a minimum [33].

**(IV) Targeting between item difficulty and participant ability:** This is established by measuring the distance between item and person means, between ceiling and floor effects and the effective operational range [43]. The effective operational range encompasses participants who have a more than 50% chance of being rated above the bottom category of the least difficult item and below the top category of the most difficult item [44]. This range is reported as a proportion of the participants’ abilities that were covered by the instrument (all items), and in this study, a range that covered 90% of the participants was considered to be highly satisfactory [45].

**(V) Rating scale functioning:** The guidelines from Linacre state the following minimum requirements: each rating scale category should include at least 10 observations; the outfit mean square (MnSq) should be below 2.0; average measures and step difficulty for each category should increase monotonically (in other words, a more difficult category should have a higher logit value); and categories should be ordered as intended, with an acceptable distance between adjacent categories (recommended distance 1.4 to 5 logits) [46].

**(VI) Differential item functioning (DIF):** This investigates the stability of item difficulty in the total dataset between participant groups (item bias) based on sex and age. DIF analysis is recommended where there are at least 100 participants per group [24]; therefore, in this study two diagnostic groups (“affective disorders” and “Attention Deficit Hyperactivity Disorder (ADHD) and autism spectrum disorders”) were included in a DIF analysis for diagnosis. Four age groups were defined and used for the DIF analyses (see Table 1). Due to the low number of older participants, the 65 + age group had fewer than 100 participants. To identify any statistically significant DIF between groups, the following two criteria were applied: 1) a difference between item measurements (DIF size) between groups of > 0.5 logits, which is large enough to have substantial consequences; and 2) a statistical significance level (*p*-value) < 0.05 [24, 47]. The analyses were performed using WINSTEPS 3.90 [48].

To explore the linear relationship between methods of calculating overall scores, Pearson’s correlation analyses were performed among three datasets with the two scoring models. These models represented the 0–100 possible range and were calculated based on the observed data as follows: (i) Missing data were imputed, and each person’s raw scores were re-calculated to an overall score on a 0–100 scale according to the IRT scoring model (WHODAS-complex model); (ii) each person’s raw scores were also summed and divided by the total available score to create an overall score on a 0–100% scale according to the simple scoring model [3] (WHODAS-simple model); and (iii) Each person’s ability measures from the Rasch analysis (in logits) were converted to a 0–100 scale in WINSTEPS (Rasch 0–100 scale). For this calculation, no imputation for missing data was performed because Rasch analysis allows for missing data. For the first two calculations, the method for imputation indicated in the WHODAS 2.0 manual was used; this specifies that, in cases where one item in a domain is missing, the mean score across all items within that domain is assigned to the missing item.

The correlation analyses were reported with the 95% confidence interval (CI) and performed using SPSS v.25 (IBM Corp, Armonk, NY).

## Results

### Validity and reliability

Of the 36 items, 97% (35 items) were within the recommended range of the infit mean square; only item D4.5 (Sexual activities) indicated a misfit (infit mean square 1.54 logits). For the outfit mean square, four items (11%) indicated misfit: D2.5 (Walking a long distance), D3.4 (Staying by yourself for a few days), D.4.5 (Sexual activities) and D6.4 (How much time did you spend on your health condition, or its consequences?). However, point-biserial correlations for the items were positive, see Table 2. Unexpected responses that caused misfit did not show shared characteristics between the respondents. In addition, these unexpected responses represented about 2% of the whole sample.

**Table 2:** Fit statistics for the Swedish 36-item version of WHODAS 2.0

Domain	Item	Item measure (logits)	n	S.E	Infit mean square	Outfit mean square	Point-biserial correlation
*D1. Understanding and communicating*							
	1. Concentrating on doing something for ten minutes	− 0.07	780	0.04	0.91	0.87	0.67
	2. Remembering to do important things	0.1	780	0.04	0.92	0.91	0.67
	3. Analysing and finding solutions to problems in day-to-day life	− 0.02	780	0.04	0.83	0.79	0.72
	4. Learning a new task, for example, learning how to get to a new place	− 0.24	780	0.04	1.00	1.01	0.63
	5. Generally understanding what people say	− 0.84	780	0.05	1.01	0.93	0.58
	6. Starting and maintaining a conversation	− 0.23	780	0.04	1.02	1.01	0.62
*D2. Getting around*							
	1. Standing for long periods such as 30 min	− 0.18	780	0.04	1.21	1.43	0.56
	2. Standing up from sitting down	− 1.05	780	0.05	1.04	1.09	0.54
	3. Moving around inside your home	− 1.38	780	0.05	0.92	0.77	0.56
	4. Getting out of your home	− 0.32	780	0.04	0.83	0.75	0.71
	**5. Walking a long distance such as a kilometre [or equivalent]?**	− 0.28	780	0.04	1.09	**1.64**	0.57
*D3. Self-care*							
	1. Washing your whole body?	− 1.19	780	0.05	0.94	0.74	0.55
	2. Getting dressed?	− 0.8	780	0.06	0.87	0.66	0.55
	3. Eating	− 0.37	780	0.04	1.14	1.17	0.56
	**4. Staying by yourself for a few days?**	− 0.04	780	0.04	1.40	**1.97**	0.50
*D4. Getting along with people*							
	1. Dealing with people you do not know?	− 0.11	780	0.04	0.95	0.91	0.66
	2. Maintaining a friendship?	− 0.07	780	0.04	0.91	0.90	0.68
	3. Getting along with people who are close to you?	− 0.48	780	0.05	0.98	0.96	0.61
	4. Making new friends?	0.5	780	0.04	1.16	1.21	0.64
	**5. Sexual activities?**	0.45	780	0.04	**1.54**	**2.24**	0.51
*D5. Life activities*							
- Sub-domain 5a: domestic responsibilities							
	1. Taking care of your household responsibilities?	0.09	780	0.04	0.82	0.77	0.73
	2. Doing most important household tasks well?	0	780	0.04	0.85	0.79	0.71
	3. Getting all the household work done that you needed to do?	0.43	780	0.04	0.88	0.87	0.72
	4. Getting your household work done as quickly as needed?	0.57	780	0.04	0.94	0.93	0.71
- Sub-domain 5b: leisure, work and school activities							
	5. Your day-to-day work/school?	0.73	652	0.04	0.85	0.82	0.75
	6. Doing your most important work/school tasks well?	0.5	648	0.04	0.83	0.77	0.75
	7. Getting all the work done that you need to do?	0.61	648	0.04	0.89	0.83	0.74
	8. Getting your work done as quickly as needed?	0.61	648	0.04	0.90	0.83	0.74
*D6. Participation in society*							
	1. How much of a problem did you have in joining in community activities (for example, festivities, religious or other activities) in the same way as anyone else can?	0.41	780	0.04	0.91	0.94	0.72
	2. How much of a problem did you have because of barriers or hindrances in the world around you?	− 0.21	780	0.04	1.08	1.00	0.60
	3. How much of a problem did you have living with dignity because of the attitudes and actions of others?	− 0.21	780	0.04	1.02	1.03	0.62
	4**. How much time did you spend on your health condition, or its consequences?**	0.73	780	0.04	1.46	**1.57**	0.48
	5. How much have you been emotionally affected by your health condition?	1.35	780	0.04	0.95	0.97	0.69
	6. How much has your health been a drain on the financial resources of you or your family?	0.33	780	0.04	1.21	1.22	0.62
	7. How much of a problem did your family have because of your health problems?	0.18	780	0.04	1.03	1.02	0.66
	8. How much of a problem did you have in doing things by yourself for relaxation or pleasure?	0.5	780	0.04	0.95	0.91	0.71

Items with misfit are in bold. (S.E. = standard error.)

Concerning dimensionality, for the whole instrument the variance explained by the measures was 48% of the total variance explained by the observations; only domain 5 (Life activities) met the recommended criteria (see Table 3). The PCA of residuals showed that the eigenvalue of the first contrast of the unexplained variance was higher than the recommended value for the whole instrument and for domain 5. This may affect the unidimensionality of the WHODAS 2.0 overall. However, the PCA supported unidimensionality of both the domain 5 sub-domains and the other domains of WHODAS 2.0. Furthermore, the point-biserial correlations were positive for all items, supporting unidimensionality by indicating that all items contributed positively to the total raw score (See Table 2). In addition, the disattenuated correlations were 1.0 or close to 1.0 between subsets of items (the domains) and 0.80 for all items in WHODAS 2.0, supporting the unidimensionality of WHODAS 2.0.

**Table 3:** Recommended and observed values of validity criteria for the Swedish WHODAS 2.0, n = 780

Criterion	Recommended value	Domain 1	Domain 2	Domain 3	Domain 4	Domain 5	*Domain 5a* *n* = *780*	*Domain 5b* *n* = *652*	Domain 6	All domains
*Unidimensionality* *(PCA of residuals)*										
Variance explained by measures	> 60%	**55**	**54**	**45**	**51**	66	77	76	**57**	**48**
Eigenvalue of the unexplained variance in 1^st^ contrast, logits	< 2.0	1.6	1.6	1.9	1.6	**3.9**	1.6	1.9	1.6	**3.8**
Disattenuated correlation	> 0.70	0.89	1	1	1	1	0.95	0.88	0.94	0.80
*Reliability*										
Cronbach’s alpha	> 0.80	0.87	0.85	**0.70**	0.82	0.93	0.94	0.95	0.88	0.96
Person reliability	> 0.80	**0.79**	**0.57**	**0.23**	**0.67**	0.86	0.90	0.91	0.83	0.91
Item reliability	> 0.80	0.98	0.99	0.99	0.99	0.99	0.99	0.96	0.99	0.99
Person separation, logits	≥ 2	**1.91**	**1.14**	**0.55**	**1.43**	2.53	2.95	3.14	2.22	3.18
Item separation, logits	≥ 2	7.99	8.15	9.75	9.68	8.44	11.60	4.70	12.56	13.08
*Targeting*										
Mean person ability, logits*	± 1	**1.91**	**2.46**	**2.17**	**1.37**	**1.20**	**2.29**	**1.57**	0.94	**1.21**
Ceiling effect	≤ 2%	**11.5**	**31.5**	**37.2**	**14.6**	**12.8**	**19.7**	**19.3**	**4.6**	**2.7**
Floor effect	≤ 2%	0	0.1	0	0	1.2	**2.2**	**7.4**	0	0
Effective operational range	≥ 90%	**83**	**58**	**52**	**73**	**78**	**70**	**69**	92	92

Deviations from the recommendation are in bold. *Reversed order of rating scale categories: low scores indicate greater difficulty

Domains: D1, Understanding and communicating; D2, Getting around; D3, Self-care; D4, Getting along with people; D5, Life activities; and D6, Participation in society. D5, Life activities, is divided in two areas: D5a = Domestic responsibilities, and D5b = Work and school. (PCA, principal component analysis.)

The items in domain 5 (Life activities) indicated the largest residual correlation between item pairs; the correlation coefficient of residuals of items D5.6 (Doing your most important work/school tasks well) and D5.7 (Getting all the work done that you need to do) was higher than the cut-off point (r = 0.72). The remaining item pairs under this domain showed residual correlations ≤ 0.65, which indicates item local independency. Residual correlations for other domains were ≤ 0.50.

Person reliability and separation values were below the recommended minimum value for domains 1–4 but above the recommended value for domain 5 (Life activities) and domain 6 (Participation in society). For the WHODAS 2.0 total score (all domains), the person reliability and separation values were above the recommended value (Cronbach’s alpha 0.91 and 3.18 logits, respectively) which indicates internal consistency between the items and the ability of the instrument to order the participants in strata based on their ability. Item reliability and separation showed high values in the WHODAS 2.0 total score (Cronbach’s alpha 0.99 and 13.08 logits, respectively) as well as in each of the domains (see Table 3).

For targeting, except for domain 6 (Participation in society), the mean of participants’ ability was more than 1.0 logit higher than the mean of the item difficulty. The proportion of participants who answered *no difficulty* (reversed to category 4) on most items (the ceiling effect) was higher than the recommended value in all domains and in the total score. Twenty-one of 780 participants (20 with affective disorders and one with psychotic disorder) reported maximum scores (*no difficulty*) on all items. The floor effect was within the recommended value for all domains and in the total score except for domains 5a (Domestic responsibilities) and 5b (Work and school) when analysed separately. No participants answered *extreme difficulty* (category 0) on all items.

For the effective operational range, WHODAS 2.0 (all domains) estimated the ability of 92% of the participants. However, the range was lower for each domain separately, see Table 3. Most of the participants outside the range had ability higher than the most difficult items. See Additional file 1: Figure S1 for the item–person map for WHODAS 2.0.

### Rating scale functioning

Regarding the rating scale, all items had more than 10 responses per rating scale category, apart from the following six items: D1.5 (Generally understanding what people say), D2.2 (Standing up from sitting down), D2.3 (Moving around inside your home), D3.1 (Washing your whole body), D3.2 (Getting dressed) and D4.3 (Getting along with people who are close to you). In these items, the number of responses for category 0 (*extreme/cannot do*) was below the recommendation; item D3.2 (Getting dressed) did not show any responses in category 0 (see Additional file 2: Table S1). For most of the items, the distance between all adjacent categories was lower than the recommended range (Table 4). In addition, for items in domains 2 (Getting around) and 3 (Self-care), category 3 (*mild*) was covered by adjacent categories, which demonstrates reversed thresholds (see Table 4 and Additional file 3: Figure S2).

**Table 4:** Rating scale category structure for all items of the Swedish WHODAS 2.0

	Value (logits) at thresholds between categories^¤^					Distance (logits) between adjacent thresholds*		
Domain	Threshold 1 (Cat. 0–1)	Threshold 2 (Cat. 1–2)	Threshold 3 (Cat. 2–3)	Threshold 4 (Cat. 3–4)	Increase monotonically	Thresholds 1–2	Thresholds 2–3	Thresholds 3–4
D1.1	− 2.16	− 0.37	0.94	1.59	Yes	− 1.79	− **1.31**	− **0.65**
D1.2	− 2.60	− 0.14	0.78	1.96	Yes	− 2.46	− **0.92**	− **1.18**
D1.3	− 2.31	− 0.27	0.83	1.74	Yes	− 2.04	− **1.10**	− **0.91**
D1.4	− 1.63	− 0.39	0.71	1.32	Yes	− **1.24**	− **1.10**	− **0.61**
D1.5	− 2.56	− 0.08	0.85	1.78	Yes	− 0.48	− **0.93**	− **0.93**
D1.6	− 1.72	− 0.15	0.67	1.21	Yes	− 1.57	− **0.82**	− **0.54**
D2.1	− 1.34	0.20	**0.59**	**0.55**	**No**	− 1.54	− **0.39**	**0.04**
D2.2	− 2.17	0.04	**1.06**	**1.06**	**No**	− 2.21	− **1.02**	**0.00**
D2.3	− 2.20	0.26	**1.01**	**0.92**	**No**	− 2.46	− **0.75**	**0.09**
D2.4	− 1.50	0.11	**0.72**	**0.66**	**No**	− 1.61	− **0.61**	**0.06**
D2.5	− 0.66	− 0.04	**0.51**	**0.18**	**No**	− **0.62**	− **0.55**	**0.33**
D3.1	− 2.01	0.38	**1.25**	**0.38**	**No**	− 2.39	− **0.87**	**0.87**
D3.2	− 0.50	**0.55**	− **0.05**	***N.A***	**No**	− **1.05**	**0.60**	***N.A***
D3.3	− 1.33	− 0.03	**0.99**	**0.37**	**No**	− **1.30**	− **0.02**	**0.62**
D3.4	− 0.66	**0.27**	**0.42**	− **0.03**	**No**	− **0.93**	− **0.15**	**0.45**
D4.1	− 1.80	0.19	0.51	1.09	Yes	− 1.99	− **0.32**	− **0.58**
D4.2	− 1.64	− 0.03	0.59	1.09	Yes	− 1.61	− **0.62**	− 0**.50**
D4.3	− 1.96	− 0.34	0.77	1.53	Yes	− 1.62	− **1.11**	− **0.76**
D4.4	− 0.97	− 0.08	0.48	0.57	Yes	− **0.89**	− **0.56**	− **0.09**
D4.5	− 0.37	− 0.17	0.19	0.34	Yes	− **0.20**	− **0.36**	− **0.15**
D5.1	− 1.74	− 0.01	0.67	1.07	Yes	− 1.73	− **0.68**	− **0.40**
D5.2	− 1.40	− 0.31	0.79	0.91	Yes	− **1.09**	− **1.10**	− **0.12**
D5.3	− 1.62	0.04	0.56	1.02	Yes	− 1.66	− **0.52**	− **0.46**
D5.4	− 1.37	0.05	0.31	1.01	Yes	− 1.42	− **0.26**	− **0.70**
D5.5	− 0.82	− 0.43	0.20	1.06	Yes	− **0.39**	− **0.63**	− **0.86**
D5.6	− 0.73	− 0.43	0.19	0.97	Yes	− **0.30**	− **0.62**	− **0.78**
D5.7	− 0.77	− 0.31	0.45	0.63	Yes	− **0.46**	− **0.76**	− **0.18**
D5.8	− 0.71	− 0.16	0.27	0.60	Yes	− **0.55**	− **0.43**	− **0.33**
D6.1	− 1.39	0.09	0.32	0.98	Yes	− 1.48	− **0.23**	− **0.66**
D6.2	− 1.39	− 0.30	0.58	1.10	Yes	− **1.09**	− **0.88**	− **0.52**
D6.3	− 1.66	0.23	0.48	0.96	Yes	− 1.89	− **0.25**	− **0.48**
D6.4	− 2.49	− 0.23	0.55	2.16	Yes	− 2.26	− **0.78**	− 1.61
D6.5	− 2.26	0.10	0.52	1.64	Yes	− 2.36	− **0.42**	− **1.12**
D6.6	− 1.26	**0.36**	**0.31**	0.59	**No**	− 1.62	**0.05**	− **0.28**
D6.7	− 2.23	0.26	0.55	1.42	Yes	− 2.49	− **0.29**	− **0.87**
D6.8	− 1.27	0.01	0.31	0.95	Yes	− **1.28**	− **0.30**	− **0.64**

NOTE: Rating scale categories are reversed: low scores indicate greater difficulty. Categories were as follows: 0 = *extreme/cannot*, 1 = *severe*, 2 = *moderate*, 3 = *mild*, 4 = *no difficulty*

Domains: D1, Understanding and communicating; D2, Getting around; D3, Self-care; D4, Getting along with people; D5, Life activities; and D6, Participation in society. D5 is divided into two sub-domains: D5a = Domestic responsibilities and D5b = Work and school

^**¤**^Values should increase monotonically; disordered thresholds are in bold

^*^The criterion for the distance between adjacent thresholds is 1.4–5.0 logits; distances not meeting the criterion are in bold

N.A. = not applicable because there were no responses in the category *extreme/cannot do*

### Differential item functioning

No DIF was found between men and women or between the diagnostic groups “affective disorders” and “ADHD and autism spectrum disorders”. However, four out of five items in domain 2 (Getting around) had significant DIF for the age group *65* + ; in other words, these items were significantly more difficult for participants in this age group than for participants in the other age groups. The fifth item in the same domain (D2.4 *Getting out of your home*) was also found to be more difficult in the age group *65* + but did not reach the threshold for significant DIF.

### Correlations between the simple and complex scoring models

A strong linear relationship was found between the different methods of calculating overall scores.

**Fig. 1:**
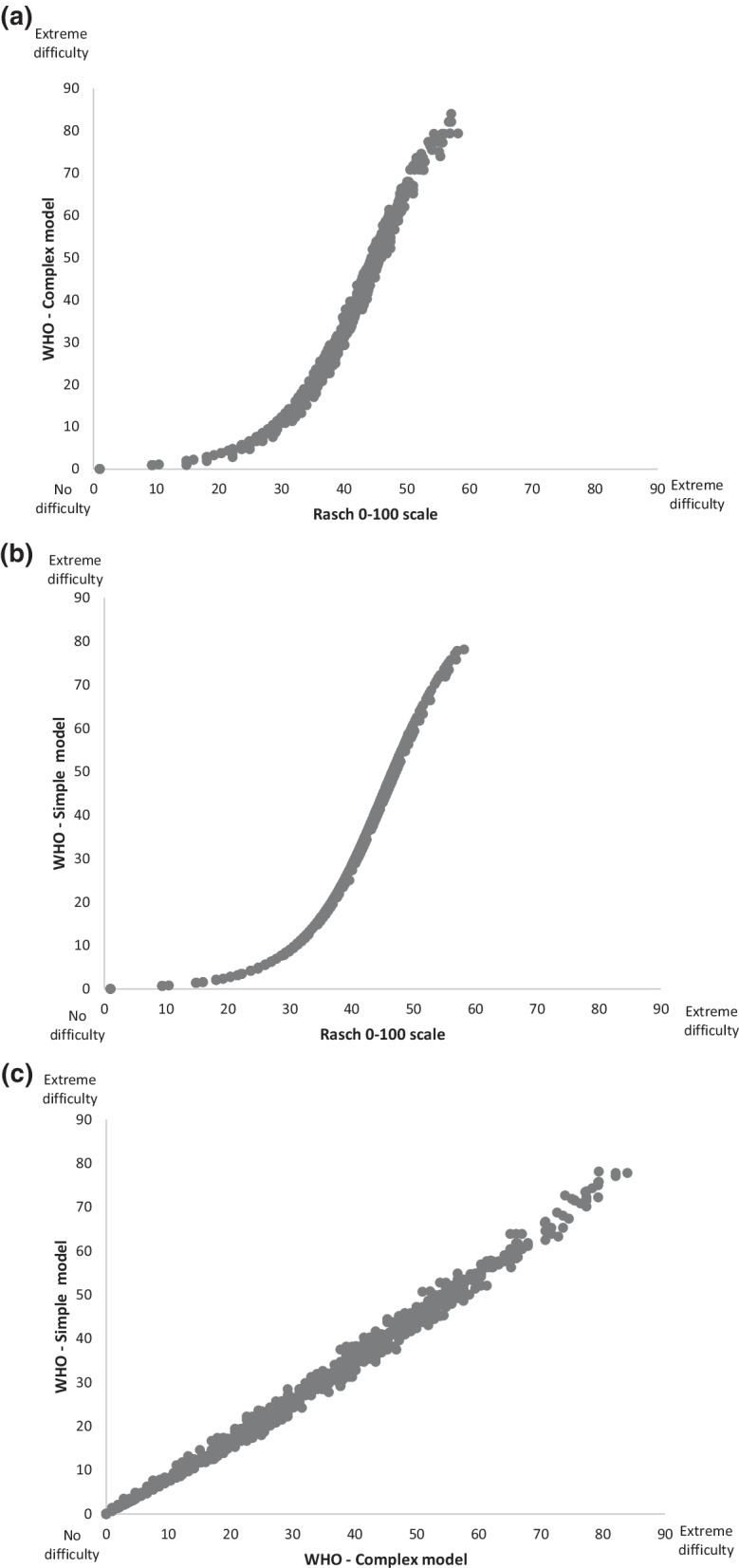
Bivariate Pearson correlation between methods for calculating an overall score on the WHODAS 2.0. All procedures transform the results to a 0–100 scale. The results are based on Swedish psychiatry outpatients (n = 780). NOTE. The Rasch 0–100 scale refers to data from new analyses where all five rating scale categories were used for each item. **a** Correlation between person measures is calculated according to the complex (IRT-based) model in the WHODAS 2.0 manual and the Rasch partial credit model (r = 0.90, *p* < 0.001). **b** Correlation between person measures is calculated according to the simple model in the WHODAS 2.0 manual (percentage of raw scores) and the Rasch partial credit model (r = 0.89, *p* < 0.001). **c** Correlation between person measures is calculated according to the simple model in the WHODAS manual (percentage of raw scores) and the complex model in the WHODAS manual (IRT-based) (r = 0.99, *p* < 0.001)

The correlation coefficient between person measures based on the WHODAS complex model and the Rasch model was 0.90 with 95% CI 0.88–0.91 (*p* < 0.001). Furthermore, the correlation coefficient between percentage of raw scores (based on the WHODAS simple model) and the Rasch model was r = 0.89 with 95% CI 0.87–0.90 (*p* < 0.001). Finally, the correlation coefficient between person measures according to percentage of raw scores (based on the simple model) and the complex model was 0.99 with 95% CI 0.995–0.996 (*p* < 0.001), see Fig. 1.

## Discussion

The results from this study contribute to building evidence for validity of the Swedish self-rated 36-item version of the WHODAS 2.0 for use in Swedish psychiatric outpatient care. The instrument’s psychometric properties contributed satisfactorily to the evidence for validity at the level of the total score. This is in line with the results of a CTT study of the Swedish version of the WHODAS 2.0 in patients with mental disorders [21]. The analyses between different methods of calculating overall scores demonstrated a high linear correlation. However, some problems were demonstrated at the domain level, and the rating scale analysis revealed problems with small distances between severity levels and disordered thresholds, which warrant revision of the rating scale categories.

Although the instrument generally fulfilled validity criteria satisfactorily, some criteria did not meet the recommended values. The 36-item WHODAS 2.0 comprises six domains, and each domain theoretically has its own dimension and construct. The total score consists of all items or the summation of all domain scores. Thus, how items, domains and the total score interact needs to be considered. Respondents who answered unexpectedly for the items with misfit did not show any common feature and represented only about 2% of the sample, which is a very low effect. In addition, deleting these responses caused the items to fit the model and no additional items with misfit were reported. Nevertheless, item D4.5 (Sexual activities) may need attention. In the construction process of the WHODAS 2.0, this item was added after the field trials on the basis of expert opinion rather than empirical evidence [4], and it has been pointed out as a problematic item in many language versions of the WHODAS II and 2.0 [14, 17, 22, 49]. Several possible explanations may account for the misfit of D4.5. Sexual activity is a sensitive topic, and asking about it could increase the risk of response bias. Park et al. considered this item as a private concern and suggested that it could be irrelevant for some people [22]. Another possible reason for the misfit in this study is that medication that enhances general functioning (such as serotonin reuptake inhibitors used for the treatment of depression) may have sexual side effects. In the first stages of the Swedish translation process, the content of item D4.5 was unclear to the respondents. When the distances between adjacent thresholds in the rating scale for item D4.5 were examined, they were all much smaller than the acceptable range, suggesting that comprehending this item, differentiating among its rating categories and giving a rating were difficult for the respondents. Rephrasing the item may be a solution; another option would be to omit it in the overall assessment of daily functioning.

The analysis of all items together indicated that they share one general dimension, namely, disability, even if the variance explained by measures was lower than recommended. This could be expected, as the six domains in WHODAS 2.0 measure different aspects of functioning. However, the point-biserial correlations were positive for all items at all domain levels and instrument levels of analysis, which was a further indication that all items positively supported the total score to reflect the general dimension. An item with negative correlation would mean that this item is not in the same dimension as the other items and does not support the unidimensionality. Moreover, the disattenuated correlation confirmed the unidimensionality even at the domain level, which may suggest that measurement error was the cause of the explained variance under the recommended value [37]. The confirmatory factor analysis in the CTT study of the Swedish version of WHODAS 2.0 indicated one general disability factor [21], which confirms the acceptable unidimensionality of the WHODAS 2.0 reported in the current study.

The fact that items in domain 5 (Life activities) indicated multidimensionality might indicate that these items cover two sub-domains: household work and workplace/school activities. This was confirmed when we divided domain 5 into two sub-domains, D5a (Domestic responsibilities) and D5b (Work and school); the proportion of the measure explained by each sub-domain increased, and the eigenvalue of the unexplained variance decreased. The local item dependency between items D5.7 and D5.8 may be explained by both items sharing the same sub-domain (5b) that is reflected in its own sub-dimension, which could be expected to indicate a high residual correlation [40]. The other high residual correlations were also between item pairs under the same sub-domains of domain 5 (D5a or D5b) and the same explanation applies.

Person reliability and separation were very low at the D1–D4 domain level, which could be explained by the low number of items in each domain, which led to an increase in error variance. However, Cronbach’s alpha values confirmed the internal consistency of items. Domain 3 (Self-care) had only four items and registered the lowest values, while domains 5 (Life activities) and 6 (Participation in society) contained eight items each and reported higher values. All items showed values close to the recommendations. Item reliability and separation were very high because of the large sample size.

Indices of targeting showed that several participants in this study had self-assessed ability in the high-functioning range. The high ceiling effect (based on the reversed rating of the categories) indicates that many patients perceived their functioning in daily life to be adequate, probably due to the sampling of relatively stable patients in outpatient units. In some cases, targeting might also be affected by response bias, because patients with certain mental disorders may have less insight. It would be interesting to study the agreement between self-reported scores from patients and proxy ratings made by a family member. Such an analysis might provide insights into the impact of the health condition on the reliability of the self-administered WHODAS 2.0. The person measures in this study indicate that the sample was mistargeted to the full range of the instrument, which was not anticipated. The participants seemed to be patients who were recovering from illness, and those who had difficulty answering the questionnaire because they had more health issues left questions unanswered and were therefore omitted from the analysis. The results suggest that physicians do not approach patients in a severe state of mental illness with a request to complete a 36-item questionnaire. A high ceiling effect of the WHODAS II and 2.0 has been shown in other studies with psychiatric populations [18, 19, 22, 50], especially in the domains of Mobility and Self-care. As indicated by the effective operational range, the total score (all WHODAS 2.0 items) seems to work better than separate domain scores for psychiatric patients; Holmberg et al. mentioned the same result in their paper on patients with psychotic disorders [50]. Participants outside of the effective operational range had higher ability, including the high ceiling effect. This may indicate that the instrument is not sensitive for measuring improvements in functioning among healthy people living in the community and people with a low degree of disability [22].

In this study, we found that the rating scale of the instrument did not perform as optimally as expected from a partial credit model on any item. Rating scale analysis indicated problems with the distance between adjacent categories of severity, especially for categories 2 (*mild*) and 3 (*moderate*). This disordering between the adjacent categories indicates that a group of participants were not able to distinguish between the meaning of the adjacent categories; especially words like “mild” and “moderate” could be used interchangeably which may lead to overestimation or underestimation of the total score and, in turn, of the disability level. Hence, attention needs to be paid to these rating scale categories, for instance, by rephrasing them to make the difference in meaning clearer or larger. Our results are supported by another study of the response categories of the 36-item version of the WHODAS 2.0, which also showed disordered thresholds for the majority of items [14]. Therefore, our recommendation for future development of the WHODAS 2.0 would be to review the rating scale and evaluate it with a larger sample and more diverse groups, including subjects with more severe mental illness.

In this study, the percentage of the raw scores calculated by the WHODAS simple model was collinear with the IRT-based WHODAS complex model and indicated a high linear relationship with the Rasch 0–100 scale. The high correlation in our study may be due to an insufficient number of scores at the extremes, especially for a high level of disability, as could be expected from our sample [51]. This is an important aspect to note when using healthcare instruments, as this finding indicates that the study needs to be replicated in other populations. However, the converted scores (IRT or Rasch 0–100) are still meaningful, since they avoid misinterpretation that may occur with the use of raw scores, especially for patients with extreme scores, and they provide a standard error for the measures (see Additional file 4: Table S2). Correlation analysis was helpful to demonstrate the relation between scoring models, However, this is not enough to allow us to recommend one scoring system over the other. We therefore recommend future studies to determine whether one scoring system discriminates more effectively between groups or is more responsive than the other.

Concerning the distribution of responses across the rating scale categories, very few or even no responses were noted in domains 2 (Getting around) and 3 (Self-care) for the “0” rating category (*extreme difficulty*). Patients with mental disorders are expected to have fewer problems with mobility (domain 2) and personal care (domain 3) and more problems with cognitive functioning, relations and participation in society. The results from this study confirm this expectation and that the instrument captures functioning overall and in the six domains, rendering it suitable for use in psychiatric outpatient care. Additionally, age-related DIF was expected on mobility items; that is, more difficulty with mobility among patients aged 65+ years was expected and confirmed.

### Study limitations

This study was conducted on a convenience sample. Hence, there was no information about the number of patients who declined to participate in the study. Potentially, the participants differ from those who declined, but we do not know in what ways. In addition, the sample was not sufficiently large to enable the analysis of DIF in all diagnostic groups. Since this study mainly included psychiatric outpatients, the WHODAS 2.0 needs to be further evaluated in a larger and more diverse sample, including inpatients and a larger number of geriatric patients, to cover the general psychiatric population.

This study is part of a number of studies on the WHODAS 2.0 in Sweden. More research is needed to establish evidence for validity of the WHODAS 2.0 for use in people with mental disorders. Since the WHODAS 2.0 has replaced the Global Assessment of Functioning as the gold standard in the DSM-5, concurrent validity between these instruments needs to be established. Future studies on the WHODAS 2.0 in patients with mental disorders should encompass a comparison of the agreement between self-reported scores from patients and those from proxy ratings by a family member, as well as experiences from the use of this instrument in clinical practice. This would be useful to further validate the WHODAS 2.0. Furthermore, comparing the 36-item and 12-item versions of the questionnaire would also be important. If the 12-item version proves to be able to estimate the level of functioning adequately, implementing this shorter version in routine clinical work would be easier. Future clinical studies also need to evaluate whether the instrument is useful for assessing additional support needs or for measuring treatment effects.

## Conclusion

We conclude that the WHODAS 2.0 fulfilled several aspects of validity and has the potential to be a useful tool in the assessment of patients with mental disorders in psychiatric outpatient practice. The instrument’s internal structure was satisfactorily valid and reliable at the level of the total score but demonstrated problems at the domain level. Rephrasing or removing item D4.5 and revising categories 2 and 3 on the rating scale for the assessment of severity are recommended improvements for the instrument; these improvements should be investigated in future studies. The WHODAS simple scoring model is easier to use in clinical practice and our results indicate that it can be used in patients with moderate psychiatric disability. The Rasch scaled scores, which are presented as a supplement to this paper (Additional file 4: Table S2), are psychometrically more precise even at low disability levels. Further investigations of different scoring models are warranted.

## Supplementary Information

**Additional file 1**.** Figure S1**. Item–person map for the Swedish 36-item WHODAS 2.0. ﻿The first column from the left orders participants based on their ability: higher is more able. Items are represented by Rasch-Thurstone thresholds between adjacent categories and the WHODAS 2.0 rating scale categories (0–4). Items are ordered based on difficulty: higher is more difficult.

**Additional file 2**.** Table S1**. Rating scale category structure for domains 2 and 3 on the Swedish 36-item WHODAS 2.0 in psychiatric patients.

**Additional file 3**.** Figure S2**. Rating scale category structure for the Swedish WHODAS 2.0 in psychiatric patients.

**Additional file 4**.** Table S2**. Conversion of total raw scores to Rasch scaled scores for the Swedish self-rated 36-item WHODAS 2.0.

## Data Availability

The datasets used and/or analysed during the current study are available from the corresponding author on reasonable request.

## Abbreviations

CTT: Classical test theory
DIF: Differential item functioning
DSM-5: Diagnostic and Statistical Manual of Mental Disorders
ICF: International Classification of Functioning, Disability and Health
IRT: Item response theory
WHODAS: World Health Organization Disability Assessment Schedule
