# Supplementary material for: Evidence for validity of the Swedish self-rated 36-item version of the World Health Organization Disability Assessment Schedule 2.0 (WHODAS 2.0) in patients with mental disorders: a multi-centre cross-sectional study using Rasch analysis
Source: J Patient Rep Outcomes. 2022 May 8;6:45. doi: 10.1186/s41687-022-00449-8 (PMC9081069; doi:10.1186/s41687-022-00449-8)
Supplement: Supplementary file 1 — Additional file 1. Figure S1. Item–person map for the Swedish 36-item WHODAS 2.0. ﻿The first column from the left orders participants based on their ability: higher is more able. Items are represented by Rasch-Thurstone thresholds between adjacent categories and the WHODAS 2.0 rating scale categories (0–4). Items are ordered based on difficulty: higher is more difficult. [file 41687_2022_449_MOESM1_ESM.pdf]

Supplementary Figure S1. Item-person map for the Swedish 36-item WHODAS 2.0

The first column from the left orders participants based on their ability, where higher is more able. The following columns order items based on their difficulty, where higher is more difficult. Each column represents a Rasch-Thurstone threshold between adjacent categories using the WHODAS 2.0 rating scale (0–4).

```
MEASURE    PERSON - MAP - ITEM - Andrich thresholds (modal categories if ordered)
               <more>|<rare>
    5          .#### +
                  .# |
                     |
                     |
                     |
                  .# |
    4              . +
                  .# |
                   . |
                  .# |
                 .## T|
                     |
    3             ## +                                D6_5  .4
                 .## |                                D6_4  .4
                 .## |
                  .# |
                .### |
                 .## S|
    2           .### +                                D1_2  .4
              .##### |                                D5_5  .4
                                            D6_5  .3
           .######## |
             .###### |          D6_5  .2              D6_8  .4
            .####### |                                D6_1  .4
                                            D6_4  .3
          ########## |T                               D5_8  .4
    1      .######## M+                               D6_6  .4
                                            D5_7  .3
          .######### |                      D1_3  .3  D6_2  .4
                                            D6_8  .3
        .########### |                      D6_1  .3  D6_3  .4
                                  D6_6  .2  D6_7  .3
       .############ |S                     D4_2  .3
                                  D6_8  .2
         .########## |                      D2_1  .3  D2_4  .4
                                            D6_3  .3
                                  D5_7  .2
        .########### |  D4_5  .1            D2_5  .3
                                  D5_1  .2
    0        ####### S+M          D1_2  .2  D2_2  .3  D3_4  .4
                                  D2_1  .2  D3_1  .3
                                  D6_3  .2
              ###### |            D4_2  .2            D2_5  .4
                        D5_8  .1
                 .## |            D1_6  .2  D2_3  .3
                                  D5_2  .2
                  .# |S           D6_2  .2            D2_3  .4
                        D4_4  .1
                   # |            D1_4  .2
                        D3_4  .1
                  .# |            D4_3  .2            D3_1  .4
                        D6_8  .1            D3_2  .3
   -1             .# +            D2_2  .2
                        D6_6  .1
                   # T|T          D2_3  .2
                        D5_3  .1
                   . |  D5_2  .1
                     |  D2_1  .1
                     |  D6_2  .1
                     |  D6_4  .1
   -2                +  D6_7  .1
                     |  D1_1  .1
                     |  D1_3  .1
                     |  D4_3  .1
                     |
                     |
   -3                +
                     |  D3_1  .1
                     |  D1_5  .1
                     |  D2_3  .1
                     |
                     |
   -4                +
               <less>|<freq>
 EACH "#" IS 5: EACH "." IS 1 TO 4
```
