# Supplementary material for: Evidence for validity of the Swedish self-rated 36-item version of the World Health Organization Disability Assessment Schedule 2.0 (WHODAS 2.0) in patients with mental disorders: a multi-centre cross-sectional study using Rasch analysis
Source: J Patient Rep Outcomes. 2022 May 8;6:45. doi: 10.1186/s41687-022-00449-8 (PMC9081069; doi:10.1186/s41687-022-00449-8)
Supplement: Supplementary file 2 — Additional file 2. Table S1. Rating scale category structure for domains 2 and 3 on the Swedish 36-item WHODAS 2.0 in psychiatric patients. [file 41687_2022_449_MOESM2_ESM.pdf]

Supplementary Table S1. Rating scale category structure for domains 2 and 3 on the Swedish 36-item WHODAS 2.0 in psychiatric patients

Domain item	Cat.	Count	Count %	Infit MnSq	Outfit MnSq	Threshold	Category measure
**Domain 2. Getting around**							
1. Standing for long periods such as 30 minutes	0	22	3	1.45	1.63	NONE	-2.76
	1	94	12	1.11	1.34	-1.34	-1.01
	2	122	16	1.15	1.25	0.2	-0.03
	3	155	20	1.22	1.92	**0.59**	0.76
	4	387	50	1.22	1.24	**0.55**	1.95
2. Standing up from sitting down	0	**2**	0	1.38	1.19	NONE	-4.39
	1	36	5	1.05	1.06	-2.17	-2.22
	2	104	13	1.04	1.16	0.04	-0.75
	3	163	21	1	1.1	**1.06**	0.25
	4	475	61	1.01	1.02	**1.06**	1.56
3. Moving around inside your home	0	**1**	0	1.54	1.49	NONE	-4.74
	1	23	3	1.07	1.1	-2.2	-2.47
	2	66	8	0.86	0.67	0.26	-1.04
	3	135	17	0.9	0.59	**1.01**	-0.12
	4	555	71	0.83	0.9	**0.92**	1.13
4. Getting out of your home	0	15	2	0.73	0.73	NONE	-3.04
	1	82	11	0.9	0.78	-1.5	-1.22
	2	128	16	0.76	0.58	0.11	-0.14
	3	159	20	0.91	0.73	**0.72**	0.7
	4	396	51	0.8	0.85	**0.66**	1.92
5. Walking a long distance such as a kilometre [or equivalent]	0	26	3	1.49	2.94	NONE	-2.34
	1	58	7	1.04	1.13	-0.66	-0.97
	2	97	12	0.99	2.21	-0.04	-0.22
	3	134	17	0.96	1.54	**0.51**	0.47
	4	465	60	0.98	0.99	**0.18**	1.57
**D3. Self-care**							
1. Washing your whole body	0	**2**	0	1.27	1.86	NONE	-4.36
	1	33	4	0.96	0.84	-2.01	-2.13
	2	72	9	0.94	0.72	0.38	-0.83
	3	98	13	0.88	0.46	**1.25**	-0.03
	4	575	74	0.89	0.93	**0.38**	1.03
2. Getting dressed	0	**0**	0	.	.	.	.
	1	18	2	0.96	0.76	NONE	-2.64
	2	59	8	0.94	0.77	-0.5	-1.22
	3	103	13	0.77	0.39	**0.55**	-0.28
	4	600	77	0.75	0.86	**-0.05**	0.88
3. Eating	0	15	2	1.39	1.74	NONE	-2.97
	1	72	9	1.16	1.19	-1.33	-1.23
	2	133	17	1.14	1.46	-0.03	-0.19
	3	129	17	1.19	0.72	**0.99**	0.61
	4	431	55	1.03	1.04	**0.37**	1.74
4. Staying by yourself for a few days	0	44	6	1.51	1.79	NONE	-2.05
	1	87	11	1.42	4.48	-0.66	-0.66
	2	93	12	1.14	1.1	**0.27**	0.04
	3	120	15	1.31	0.88	**0.42**	0.66
	4	436	56	1.41	1.44	**-0.03**	1.67

Reversed thresholds in bold.
Note: Order of categories 0–4 is reversed as follows: 0=extreme/cannot do, 1=severe, 2=moderate, 3=mild and 4=no difficulty. (Cat.= category; MnSq = mean of the squared residuals.)
