# Supplementary material for: Evidence for validity of the Swedish self-rated 36-item version of the World Health Organization Disability Assessment Schedule 2.0 (WHODAS 2.0) in patients with mental disorders: a multi-centre cross-sectional study using Rasch analysis
Source: J Patient Rep Outcomes. 2022 May 8;6:45. doi: 10.1186/s41687-022-00449-8 (PMC9081069; doi:10.1186/s41687-022-00449-8)
Supplement: Supplementary file 3 — Additional file 3. Figure S2. Rating scale category structure for the Swedish WHODAS 2.0 in psychiatric patients. [file 41687_2022_449_MOESM3_ESM.pdf]

Supplementary Figure S2. Rating scale category structure for the Swedish WHODAS 2.0 in psychiatric patients

NOTE: The order of rating scale categories is reversed as follows: 0 = *extreme/cannot* (red), 1 = *severe* (blue), 2 = *moderate* (pink), 3 = *mild* (black), and 4 = *no difficulty* (green).

D1_1 refers to the first item in domain 1; D2_1 refers to the first item in domain 2; and so on.

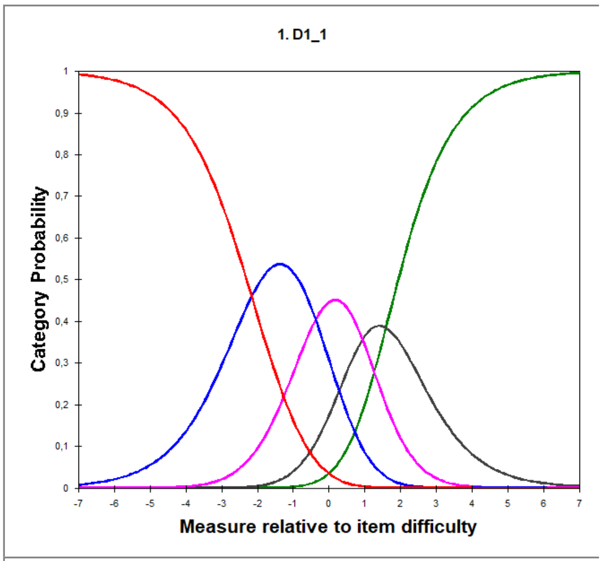
1. D1_1
Category Probability
1
0,9
0,8
0,7
0,6
0,5
0,4
0,3
0,2
0,1
0
-7 -6 -5 -4 -3 -2 -1 0 1 2 3 4 5 6 7
Measure relative to item difficulty

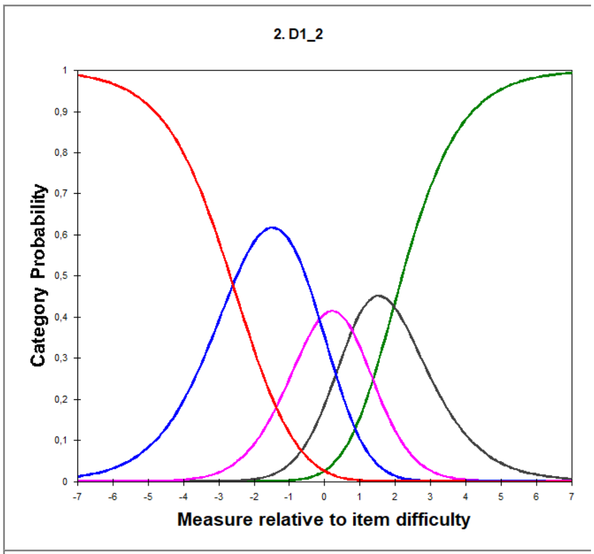
2. D1_2
Category Probability
1
0,9
0,8
0,7
0,6
0,5
0,4
0,3
0,2
0,1
0
-7 -6 -5 -4 -3 -2 -1 0 1 2 3 4 5 6 7
Measure relative to item difficulty

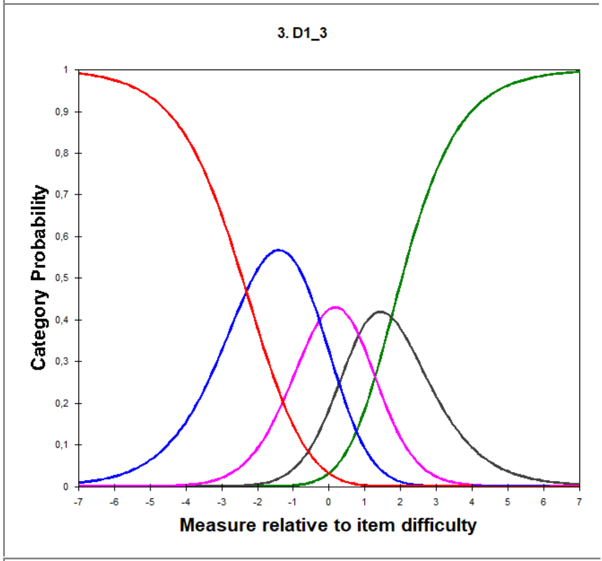
3. D1_3
Category Probability
1
0,9
0,8
0,7
0,6
0,5
0,4
0,3
0,2
0,1
0
-7 -6 -5 -4 -3 -2 -1 0 1 2 3 4 5 6 7
Measure relative to item difficulty

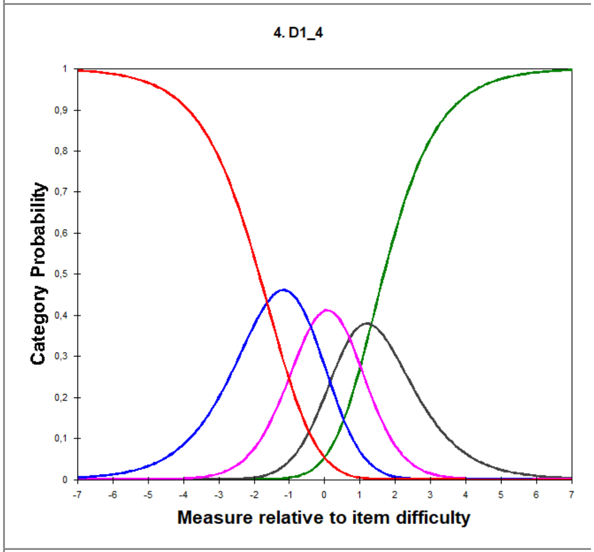
4. D1_4
Category Probability
1
0,9
0,8
0,7
0,6
0,5
0,4
0,3
0,2
0,1
0
-7 -6 -5 -4 -3 -2 -1 0 1 2 3 4 5 6 7
Measure relative to item difficulty

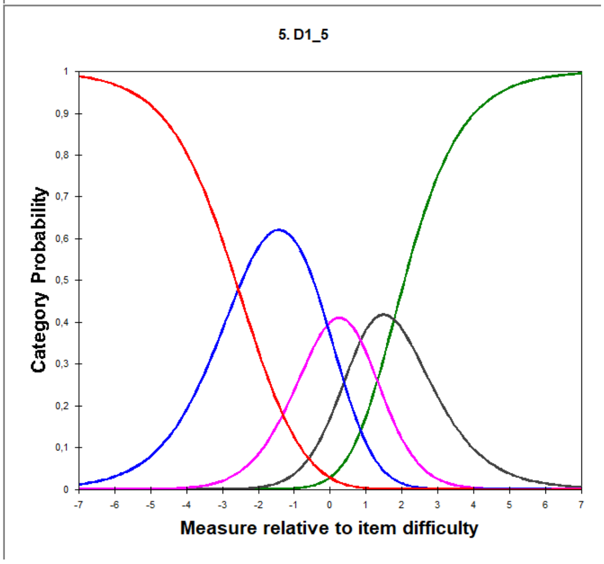
5. D1_5
Category Probability
1
0,9
0,8
0,7
0,6
0,5
0,4
0,3
0,2
0,1
0
-7 -6 -5 -4 -3 -2 -1 0 1 2 3 4 5 6 7
Measure relative to item difficulty

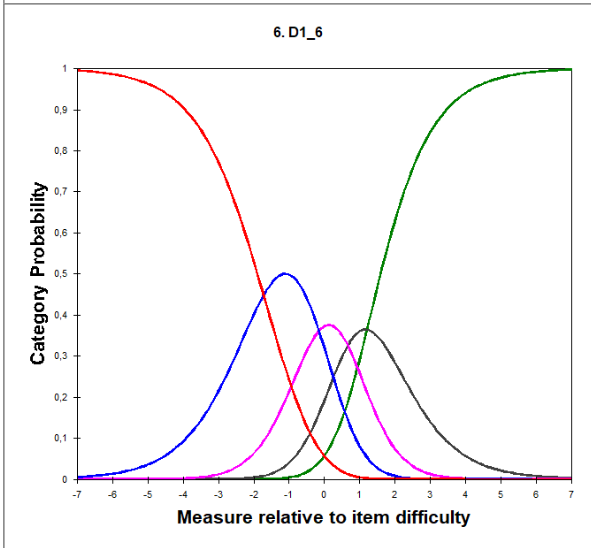
6. D1_6
Category Probability
1
0,9
0,8
0,7
0,6
0,5
0,4
0,3
0,2
0,1
0
-7 -6 -5 -4 -3 -2 -1 0 1 2 3 4 5 6 7
Measure relative to item difficulty

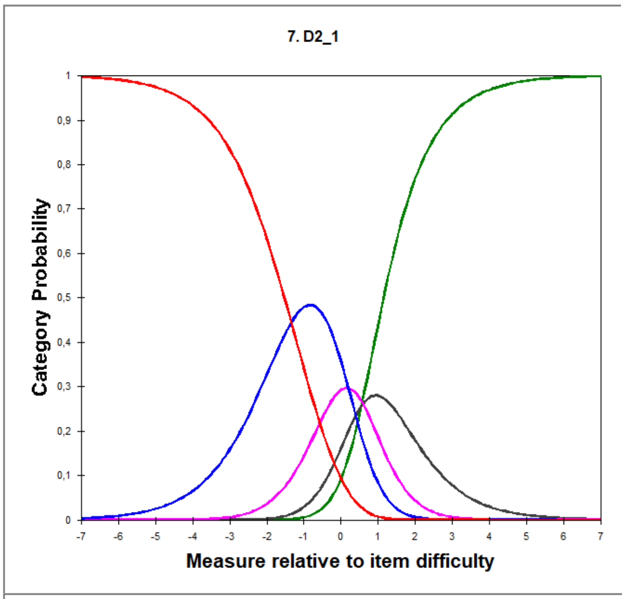
7. D2_1
Category Probability
Measure relative to item difficulty

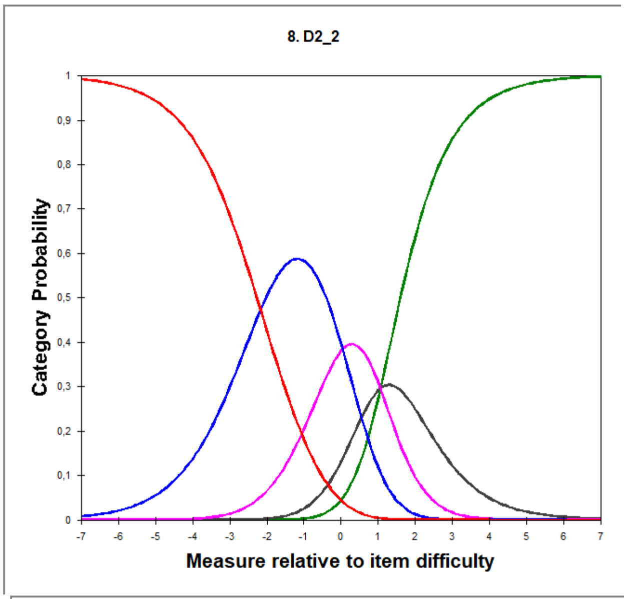
8. D2_2
Category Probability
Measure relative to item difficulty

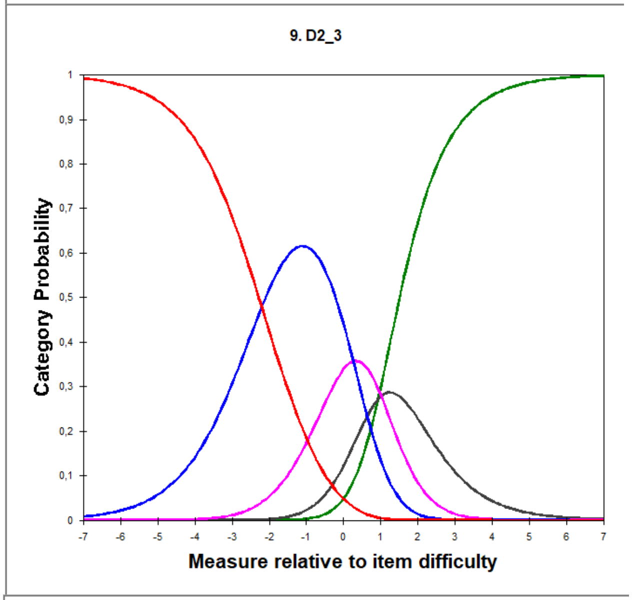
9. D2_3
Category Probability
Measure relative to item difficulty

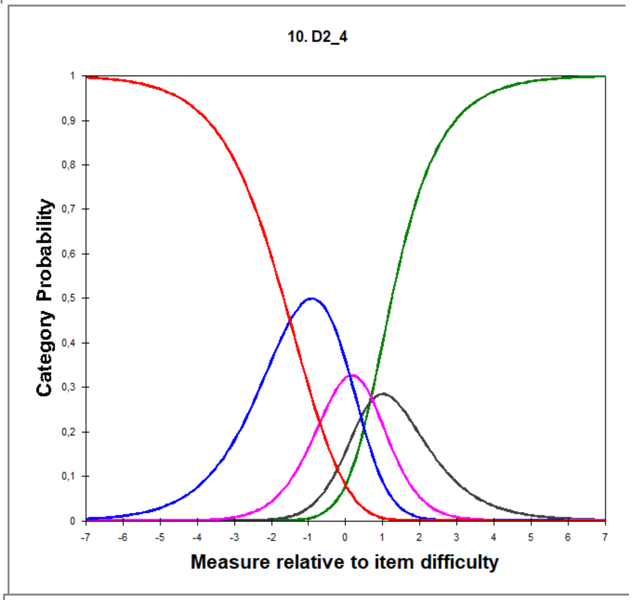
10. D2_4
Category Probability
Measure relative to item difficulty

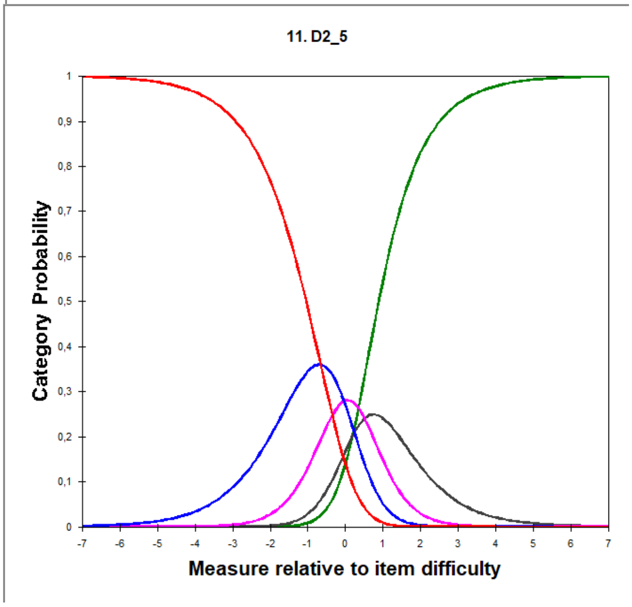
11. D2_5
Category Probability
Measure relative to item difficulty

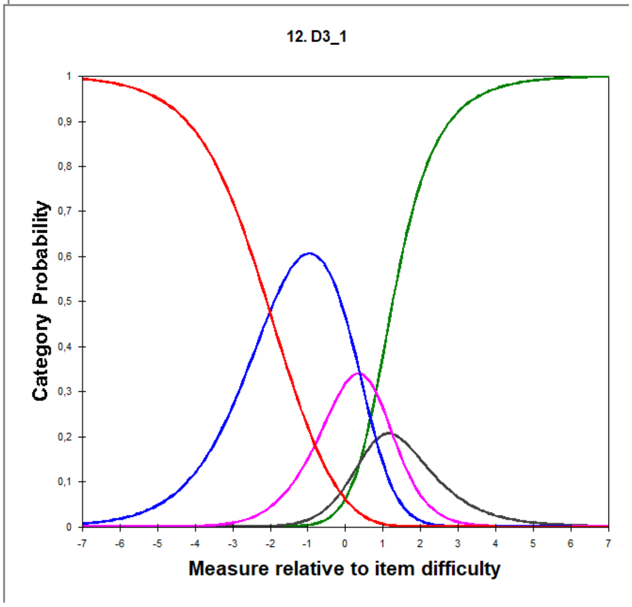
12. D3_1
Category Probability
Measure relative to item difficulty

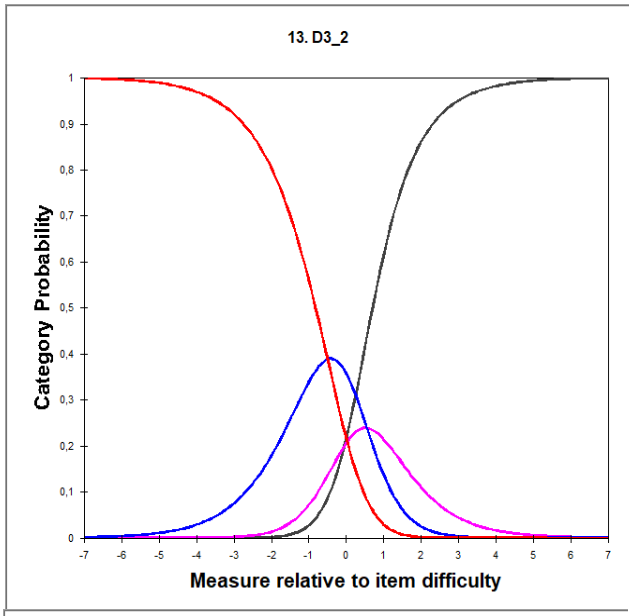
13. D3_2
Category Probability
Measure relative to item difficulty

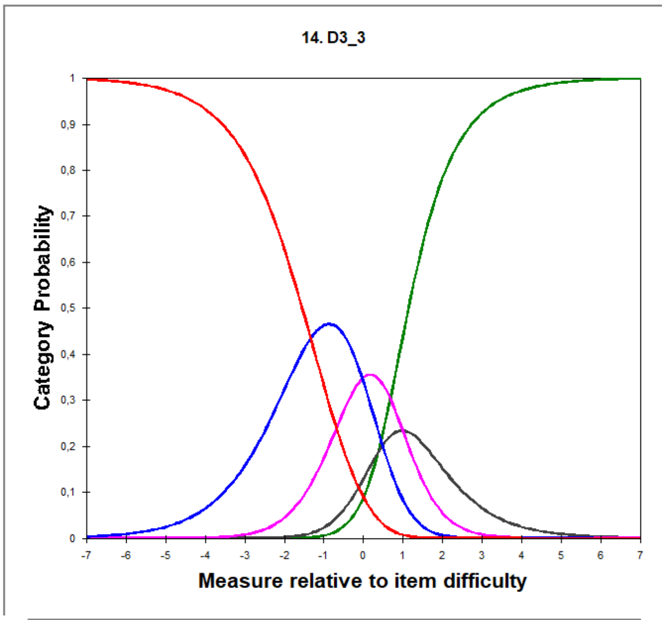
14. D3_3
Category Probability
Measure relative to item difficulty

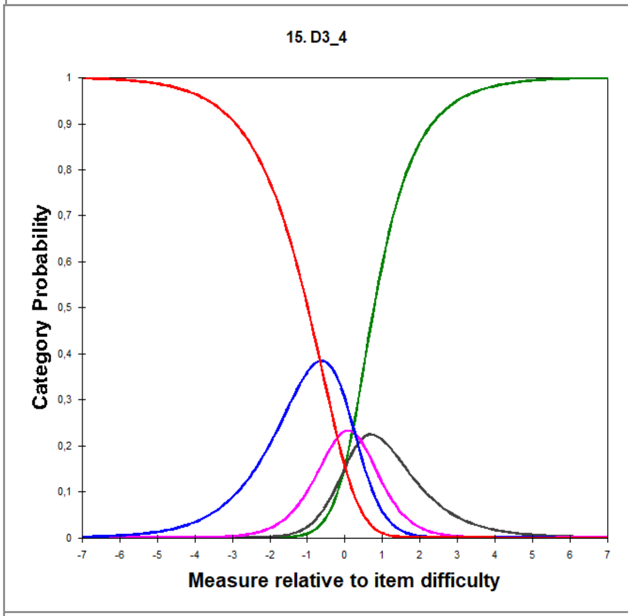
15. D3_4
Category Probability
Measure relative to item difficulty

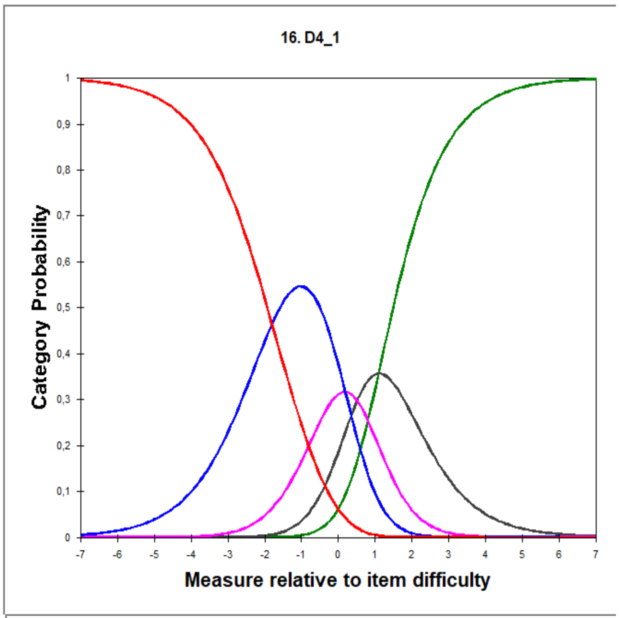
16. D4_1
Category Probability
Measure relative to item difficulty

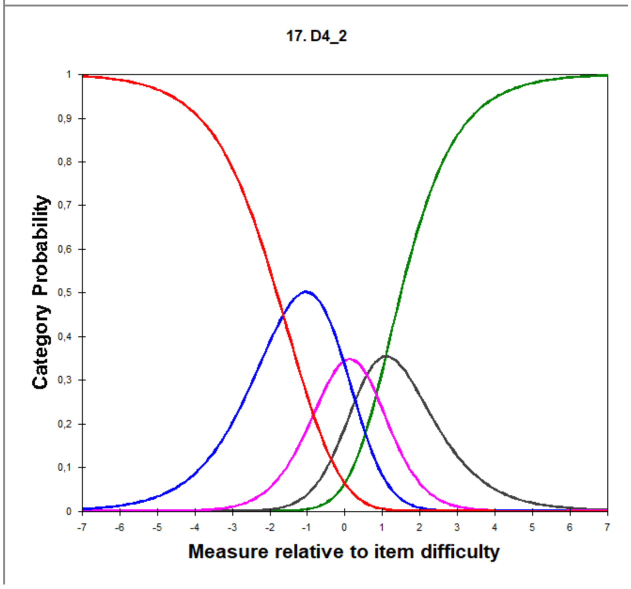
17. D4_2
Category Probability
Measure relative to item difficulty

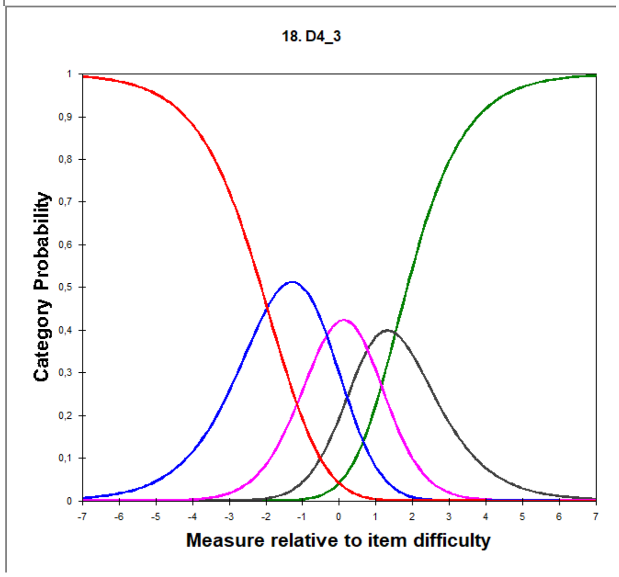
18. D4_3
Category Probability
Measure relative to item difficulty

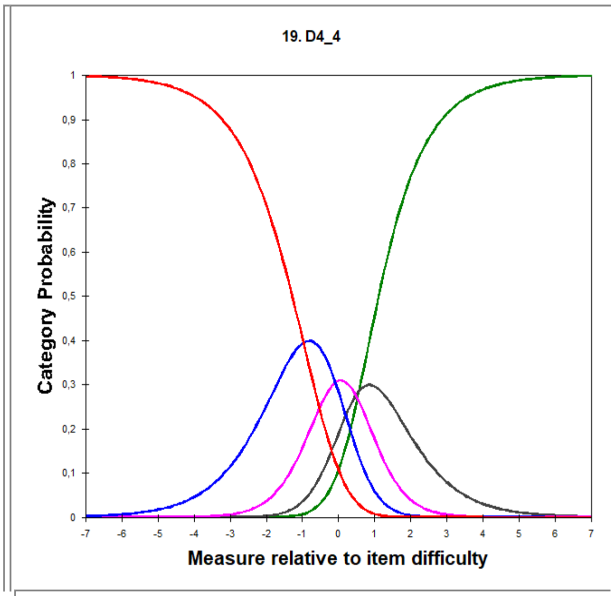
19. D4_4
Category Probability
1
0,9
0,8
0,7
0,6
0,5
0,4
0,3
0,2
0,1
0
-7 -6 -5 -4 -3 -2 -1 0 1 2 3 4 5 6 7
Measure relative to item difficulty

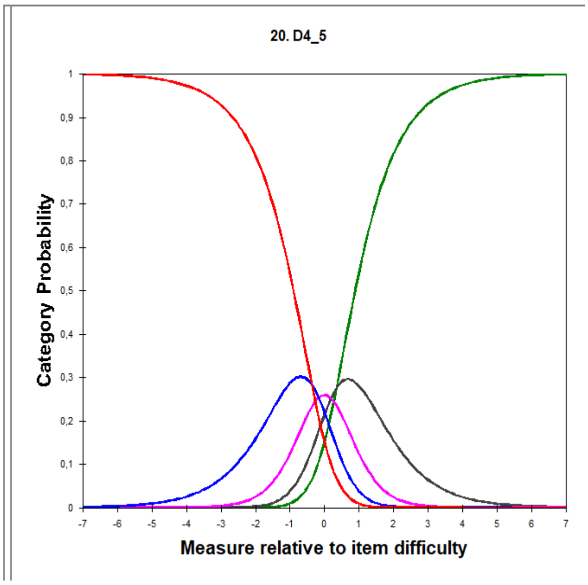
20. D4_5
Category Probability
1
0,9
0,8
0,7
0,6
0,5
0,4
0,3
0,2
0,1
0
-7 -6 -5 -4 -3 -2 -1 0 1 2 3 4 5 6 7
Measure relative to item difficulty

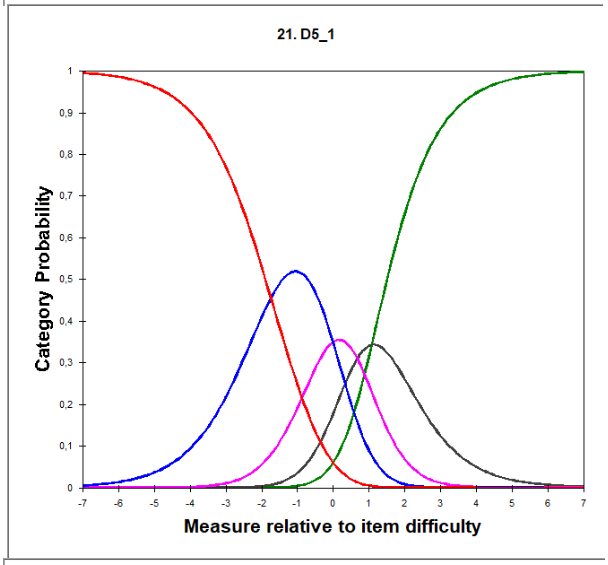
21. D5_1
Category Probability
1
0,9
0,8
0,7
0,6
0,5
0,4
0,3
0,2
0,1
0
-7 -6 -5 -4 -3 -2 -1 0 1 2 3 4 5 6 7
Measure relative to item difficulty

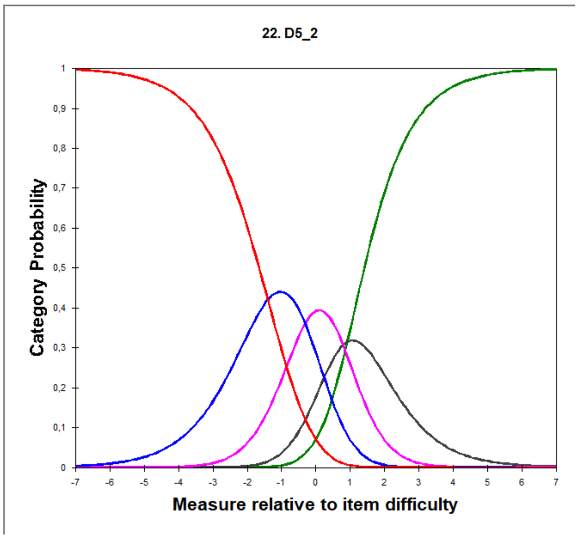
22. D5_2
Category Probability
1
0,9
0,8
0,7
0,6
0,5
0,4
0,3
0,2
0,1
0
-7 -6 -5 -4 -3 -2 -1 0 1 2 3 4 5 6 7
Measure relative to item difficulty

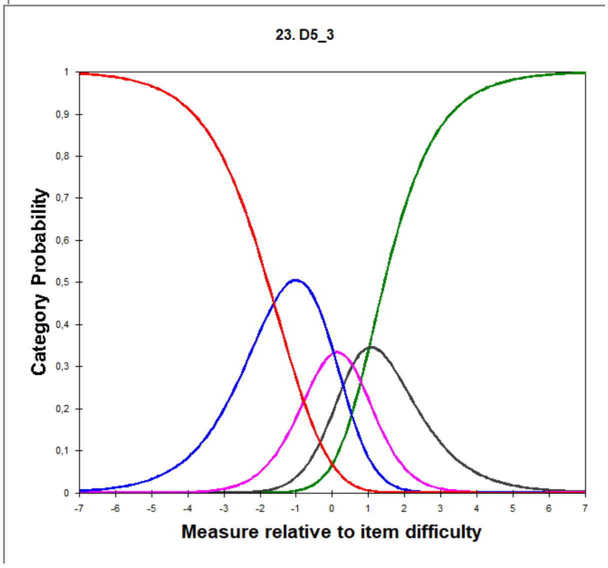
23. D5_3
Category Probability
1
0,9
0,8
0,7
0,6
0,5
0,4
0,3
0,2
0,1
0
-7 -6 -5 -4 -3 -2 -1 0 1 2 3 4 5 6 7
Measure relative to item difficulty

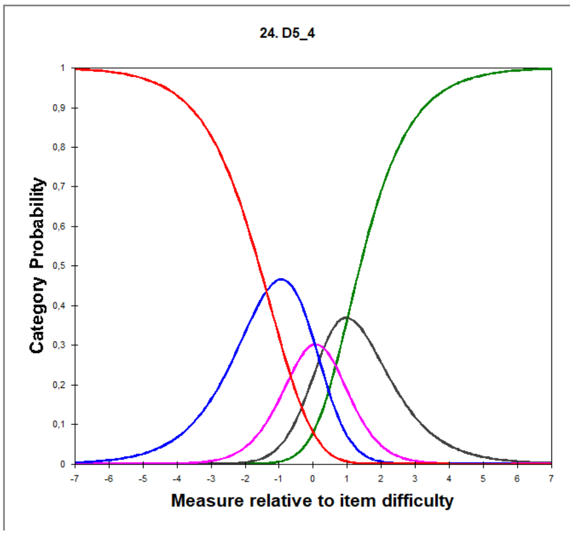
24. D5_4
Category Probability
1
0,9
0,8
0,7
0,6
0,5
0,4
0,3
0,2
0,1
0
-7 -6 -5 -4 -3 -2 -1 0 1 2 3 4 5 6 7
Measure relative to item difficulty

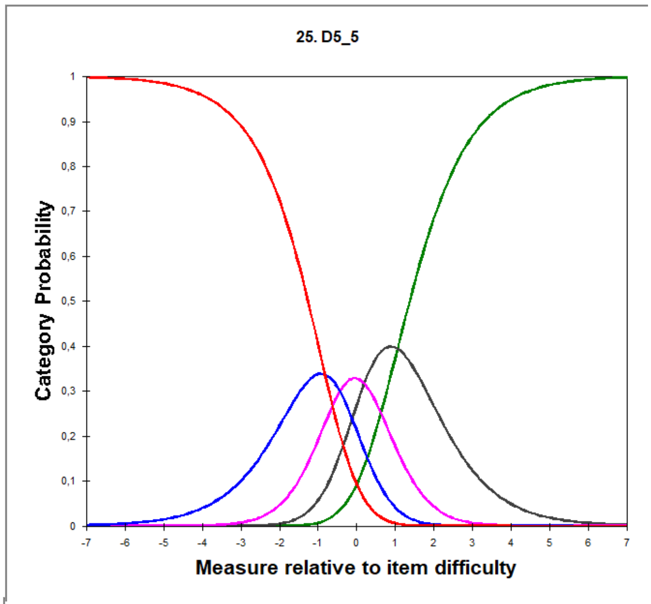
25. D5_5
Category Probability
1 0,9 0,8 0,7 0,6 0,5 0,4 0,3 0,2 0,1 0
-7 -6 -5 -4 -3 -2 -1 0 1 2 3 4 5 6 7
Measure relative to item difficulty

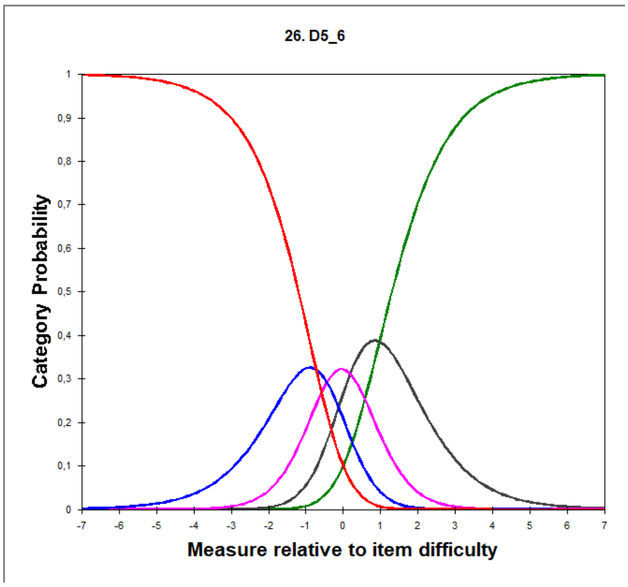
26. D5_6
Category Probability
1 0,9 0,8 0,7 0,6 0,5 0,4 0,3 0,2 0,1 0
-7 -6 -5 -4 -3 -2 -1 0 1 2 3 4 5 6 7
Measure relative to item difficulty

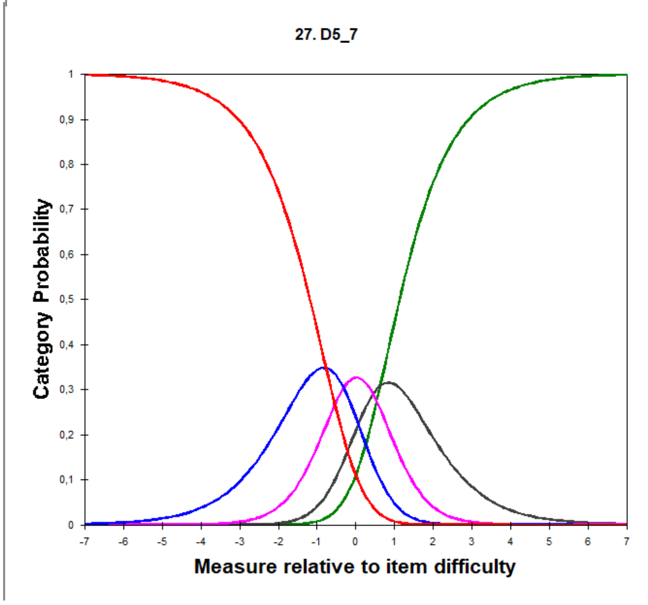
27. D5_7
Category Probability
1 0,9 0,8 0,7 0,6 0,5 0,4 0,3 0,2 0,1 0
-7 -6 -5 -4 -3 -2 -1 0 1 2 3 4 5 6 7
Measure relative to item difficulty

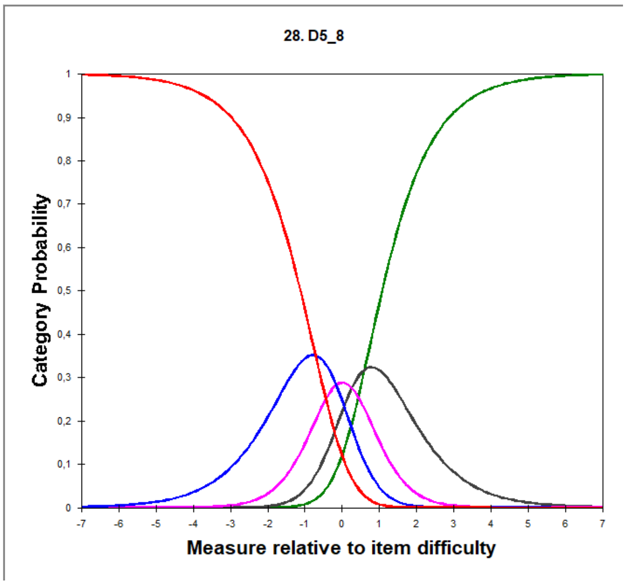
28. D5_8
Category Probability
1 0,9 0,8 0,7 0,6 0,5 0,4 0,3 0,2 0,1 0
-7 -6 -5 -4 -3 -2 -1 0 1 2 3 4 5 6 7
Measure relative to item difficulty

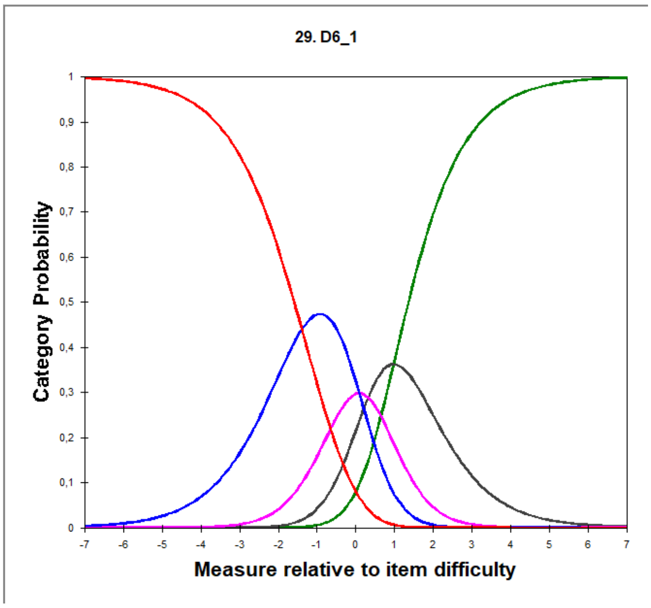
29. D6_1
Category Probability
1 0,9 0,8 0,7 0,6 0,5 0,4 0,3 0,2 0,1 0
-7 -6 -5 -4 -3 -2 -1 0 1 2 3 4 5 6 7
Measure relative to item difficulty

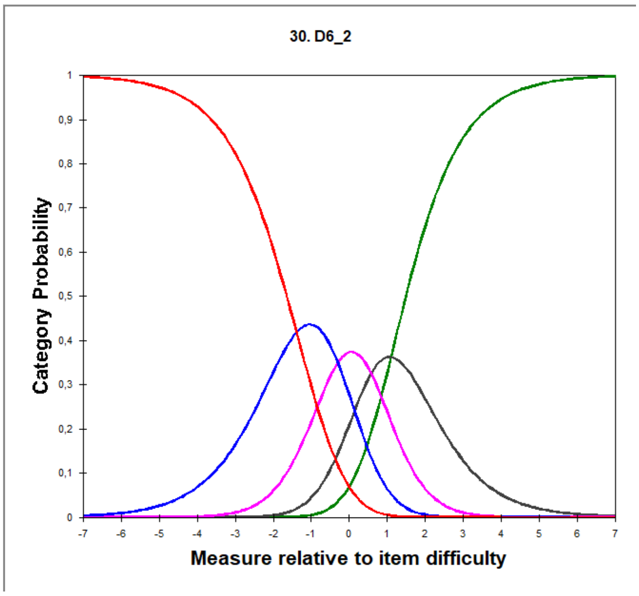
30. D6_2
Category Probability
1 0,9 0,8 0,7 0,6 0,5 0,4 0,3 0,2 0,1 0
-7 -6 -5 -4 -3 -2 -1 0 1 2 3 4 5 6 7
Measure relative to item difficulty

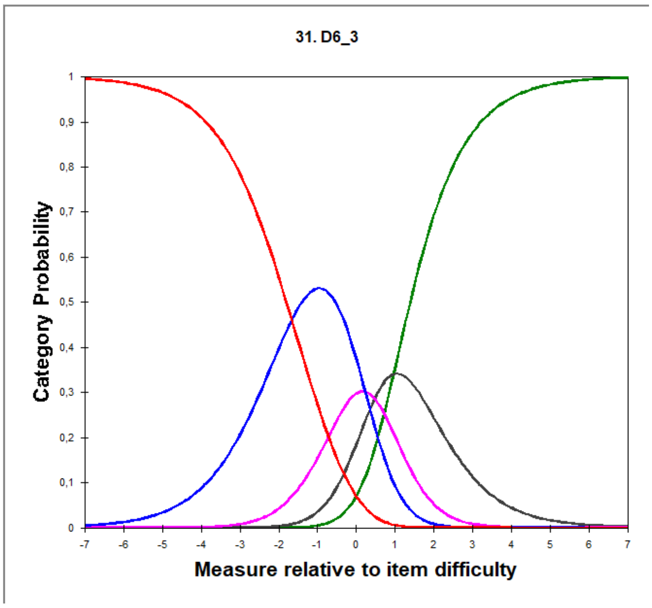
31. D6_3
Category Probability
Measure relative to item difficulty

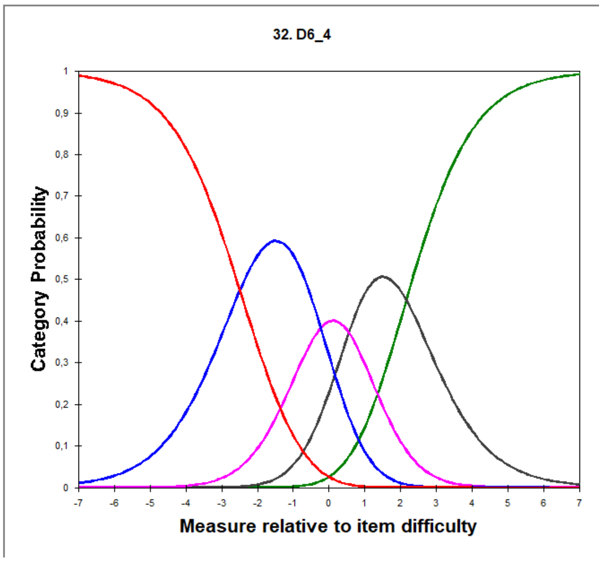
32. D6_4
Category Probability
Measure relative to item difficulty

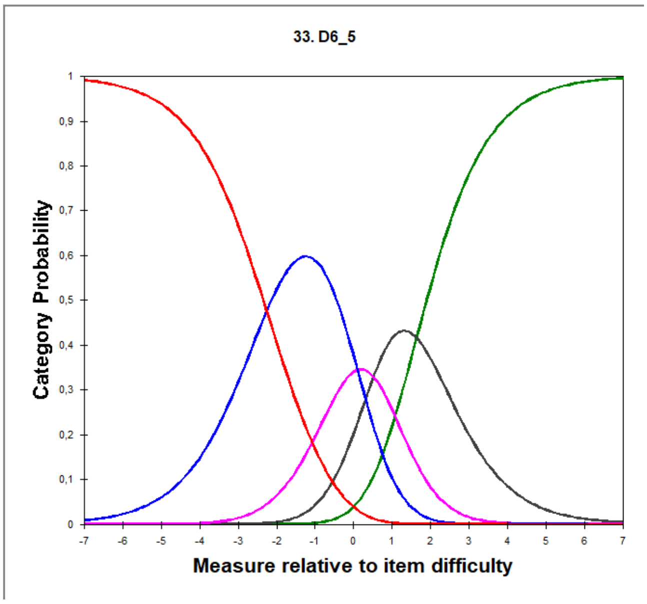
33. D6_5
Category Probability
Measure relative to item difficulty

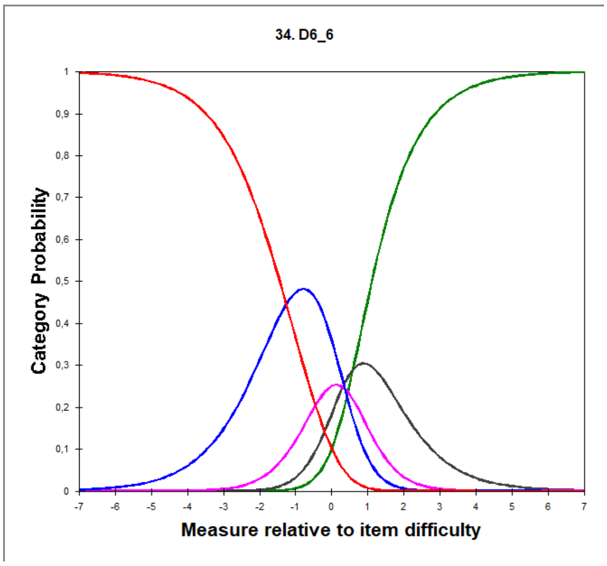
34. D6_6
Category Probability
Measure relative to item difficulty

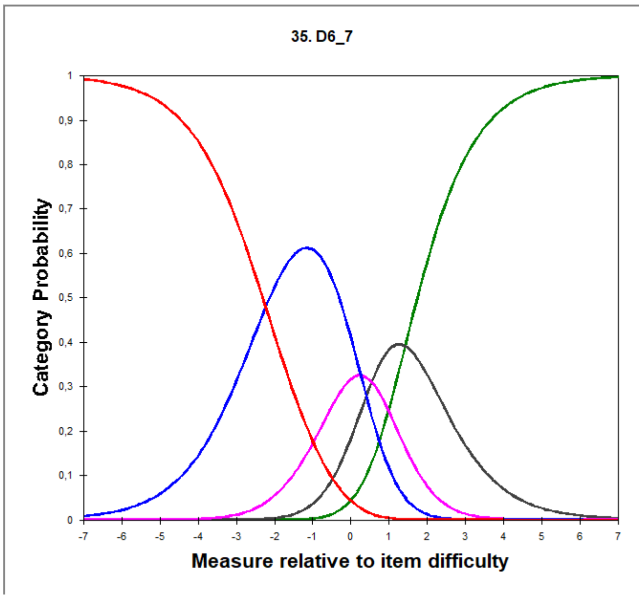
35. D6_7
Category Probability
Measure relative to item difficulty

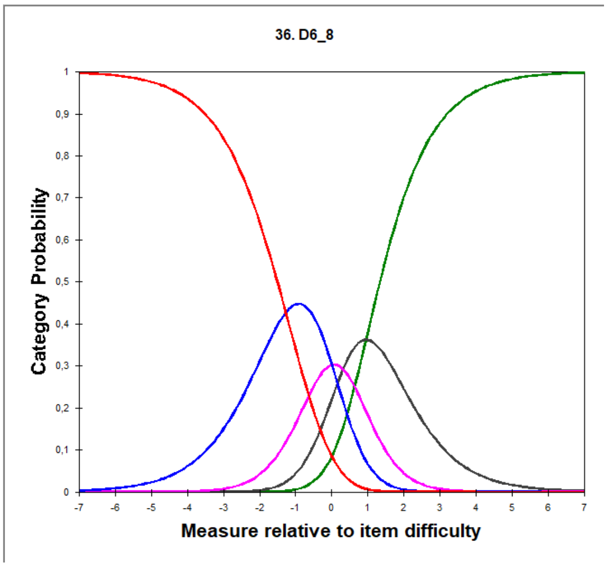
36. D6_8
Category Probability
Measure relative to item difficulty
