# Supplementary material for: Evidence for validity of the Swedish self-rated 36-item version of the World Health Organization Disability Assessment Schedule 2.0 (WHODAS 2.0) in patients with mental disorders: a multi-centre cross-sectional study using Rasch analysis
Source: J Patient Rep Outcomes. 2022 May 8;6:45. doi: 10.1186/s41687-022-00449-8 (PMC9081069; doi:10.1186/s41687-022-00449-8)
Supplement: Supplementary file 4 — Additional file 4. Table S2. Conversion of total raw scores to Rasch scaled scores for the Swedish self-rated 36-item WHODAS 2.0. [file 41687_2022_449_MOESM4_ESM.pdf]

Supplementary Table S2. Conversion of total raw scores to Rasch scaled scores for the Swedish self-rated 36-item WHODAS 2.0

Total raw score	Scaled score	S.E.	Total raw score	Scaled score	S.E.	Total raw score	Scaled score	S.E.
0	0.00	14.19	48	41.64	1.30	96	52.06	1.43
1	9.42	7.81	49	41.86	1.30	97	52.33	1.44
2	14.89	5.55	50	42.08	1.29	98	52.60	1.46
3	18.13	4.55	51	42.29	1.29	99	52.87	1.47
4	20.43	3.95	52	42.50	1.28	100	53.15	1.48
5	22.23	3.54	53	42.71	1.28	101	53.44	1.50
6	23.71	3.24	54	42.92	1.27	102	53.73	1.52
7	24.96	3.00	55	43.13	1.27	103	54.03	1.53
8	26.05	2.81	56	43.34	1.27	104	54.34	1.55
9	27.01	2.65	57	43.55	1.26	105	54.65	1.57
10	27.87	2.52	58	43.75	1.26	106	54.97	1.59
11	28.66	2.41	59	43.96	1.26	107	55.30	1.61
12	29.37	2.31	60	44.16	1.26	108	55.64	1.63
13	30.03	2.22	61	44.36	1.26	109	55.99	1.65
14	30.65	2.14	62	44.57	1.25	110	56.34	1.67
15	31.22	2.07	63	44.77	1.25	111	56.71	1.70
16	31.76	2.01	64	44.97	1.25	112	57.08	1.72
17	32.26	1.95	65	45.17	1.25	113	57.47	1.75
18	32.74	1.90	66	45.38	1.25	114	57.87	1.78
19	33.20	1.86	67	45.58	1.25	115	58.29	1.81
20	33.63	1.81	68	45.78	1.26	116	58.72	1.84
21	34.05	1.77	69	45.99	1.26	117	59.16	1.87
22	34.44	1.74	70	46.19	1.26	118	59.62	1.90
23	34.82	1.70	71	46.40	1.26	119	60.09	1.94
24	35.19	1.67	72	46.60	1.26	120	60.59	1.98
25	35.54	1.64	73	46.81	1.27	121	61.10	2.02
26	35.88	1.61	74	47.01	1.27	122	61.64	2.06
27	36.21	1.59	75	47.22	1.27	123	62.20	2.11
28	36.53	1.56	76	47.43	1.28	124	62.78	2.15
29	36.84	1.54	77	47.64	1.28	125	63.39	2.21
30	37.15	1.52	78	47.85	1.28	126	64.04	2.26
31	37.44	1.50	79	48.07	1.29	127	64.71	2.32
32	37.73	1.48	80	48.28	1.29	128	65.43	2.39
33	38.01	1.46	81	48.50	1.30	129	66.18	2.46
34	38.28	1.45	82	48.72	1.30	130	66.99	2.54
35	38.55	1.43	83	48.94	1.31	131	67.85	2.62
36	38.81	1.42	84	49.16	1.32	132	68.77	2.72
37	39.07	1.41	85	49.39	1.32	133	69.76	2.83
38	39.32	1.39	86	49.61	1.33	134	70.84	2.96
39	39.57	1.38	87	49.84	1.34	135	72.03	3.11
40	39.81	1.37	88	50.08	1.35	136	73.35	3.29
41	40.05	1.36	89	50.31	1.36	137	74.84	3.52
42	40.29	1.35	90	50.55	1.37	138	76.57	3.80
43	40.52	1.34	91	50.79	1.37	139	78.62	4.19
44	40.75	1.33	92	51.04	1.38	140	81.18	4.76
45	40.98	1.32	93	51.29	1.40	141	84.67	5.73
46	41.20	1.32	94	51.54	1.41	142	90.41	7.93
47	41.42	1.31	95	51.80	1.42	143	100.00	14.26
